# Genome-mining-guided discovery of coumarubrin: A novel aminocoumarin-substituted rubromycin antibiotic

**DOI:** 10.1093/jimb/kuaf018

**Published:** 2025-07-04

**Authors:** Heiner G Weddeling, Sven T Sowa, Selina Bosshardt, Lukas Schwimbersky, Malik Rakhmanov, Robin Teufel

**Affiliations:** Pharmaceutical Biology, Department of Pharmaceutical Sciences, University of Basel, Klingelbergstrasse 50, 4056 Basel, Switzerland; Pharmaceutical Biology, Department of Pharmaceutical Sciences, University of Basel, Klingelbergstrasse 50, 4056 Basel, Switzerland; Pharmaceutical Biology, Department of Pharmaceutical Sciences, University of Basel, Klingelbergstrasse 50, 4056 Basel, Switzerland; Pharmaceutical Biology, Department of Pharmaceutical Sciences, University of Basel, Klingelbergstrasse 50, 4056 Basel, Switzerland; Pharmaceutical Biology, Department of Pharmaceutical Sciences, University of Basel, Klingelbergstrasse 50, 4056 Basel, Switzerland; Pharmaceutical Biology, Department of Pharmaceutical Sciences, University of Basel, Klingelbergstrasse 50, 4056 Basel, Switzerland

**Keywords:** Griseorhodins, Rubromycins, Pentangular aromatic polyketides, Aminocoumarin antibiotics, Actinobacteria

## Abstract

Rubromycins are bacterial aromatic polyketides containing a hallmark spiroketal pharmacophore produced by type II polyketide synthases and accessory enzymes. They generally display cytotoxic and antimicrobial properties, frequently disrupting cellular processes and proteins associated with nucleic acids, such as DNA helicase or telomerase. Among the known rubromycin congeners, hyaluromycin stands out due to a 2-amino-3-hydroxycyclopent-2-enone (C_5_N) substitution that is presumably installed by an amide bond synthetase (ABS). Here, we used bioinformatic analysis to identify uncharacterized biosynthetic gene clusters and potential rubromycin producer strains encoding putative ABSs but lacking the enzymes responsible for C_5_N formation, suggesting potentially novel substituents. One of these strains, *Lentzea tibetensis*, was successfully cultivated and confirmed to produce a previously undescribed aminocoumarin-substituted rubromycin polyketide, named coumarubrin, as verified by high-resolution mass spectrometry (HRMS) and comprehensive nuclear magnetic resonance (NMR) spectroscopy. Electronic circular dichroism spectroscopy indicates an absolute configuration identical to that of previously characterized rubromycins, while the first bioactivity assays demonstrated potent inhibitory activity against Gram-positive bacteria.

**One-Sentence Summary**: This study reports the discovery of a novel member of the rubromycins, antibiotic and cytotoxic aromatic polyketides produced by Actinobacteria, which is fused to a distinct aminocoumarin moiety.

## Graphical Abstract

**Graphical Abstract:**
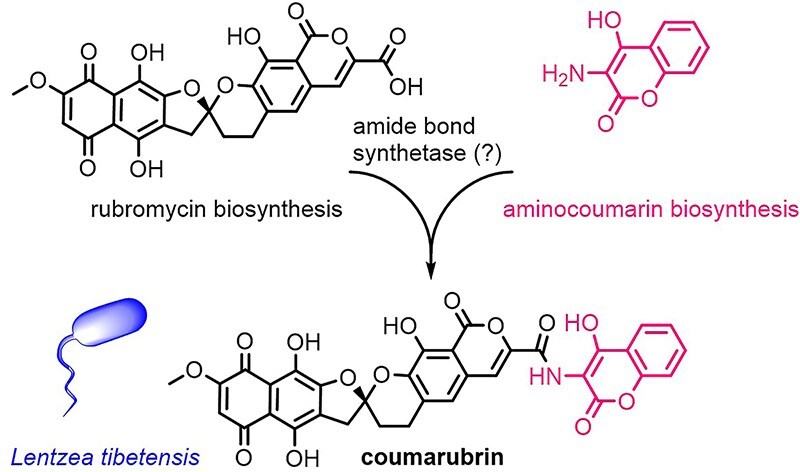

## Introduction

**Fig. 1.**
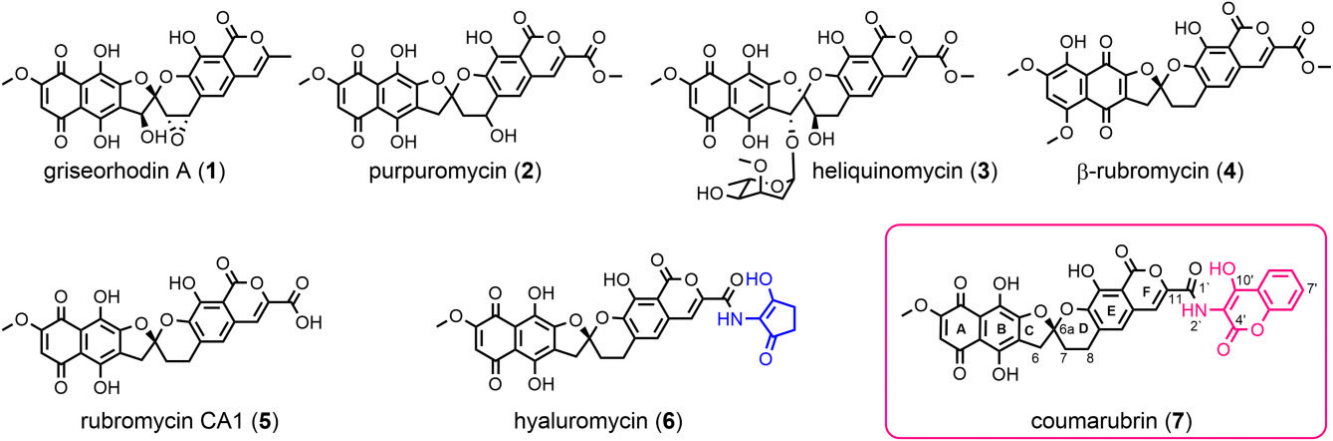
Overview of selected rubromycin polyketides. The natural product identified in this study, coumarubrin (**7**), is highlighted in a red box and features a rubromycin CA1 (**5**) backbone connected at C1’ to a distinct aminocoumarin moiety (in red), which is presumably attached by a pathway-specific amide bond synthetase similar to the C_5_N ring (in blue) of hyaluromycin (**6**). The spiroketal configuration of **7** matches that of other rubromycins, as confirmed by ECD measurements.

Pentangular aromatic polyketides such as the benastatins, fredericamycins or the griseorhodins/rubromycins are among the largest polyphenols produced by type II polyketide synthases (PKSs) in Actinobacteria (Lackner et al., 2007). In case of the griseorhodins/rubromycins, the two central rings of the pentangular backbone are further modified into the characteristic bisbenzannulated [5,6]-spiroketal pharmacophore by extensive tailoring reactions involving predominantly flavoenzymes (Frensch et al., 2021; Toplak & Teufel, 2022; Toplak et al., 2022; Toplak, Matthews, et al., 2021; Toplak, Saleem-Batcha, et al., 2021 [see Fig. S1 for a schematic overview of the rubromycin biosynthetic pathway]). The resulting structures feature highly oxidized western naphthazarin and eastern isocoumarin molecule halves connected via the central spiroketal. Rubromycins demonstrate potent antimicrobial and anticancer properties, as well as inhibitory effects on enzymes that mostly interact with nucleic acids, such as HIV reverse transcriptase, DNA helicase or human telomerase (Atkinson & Brimble, 2015; Brasholz et al., 2007; Ueno et al., 2000). The core structure of these pentangular aromatic polyketides is built from 13 C_2_-units that are condensed by the minimal PKS complex. The resulting C_26_ polyketide is assumed to serve as a universal precursor for multiple classes of pentangular polyketides, including the fredericamycins, benestatins, and xantholipins (Hertweck et al., 2007; Lackner et al., 2007). In the case of rubromycins, the initial scaffold is further processed by a unique set of flavin-dependent oxygenases and oxidases to form the spiroketal moiety (Frensch et al., 2021; Li & Piel, 2002; Toplak, Saleem-Batcha, et al., 2021; Yunt et al., 2009). Until today, 21 rubromycin-type polyketide natural products have been isolated, for example griseorhodin A (**1**), purpuromycin (**2**), heliquinomycin (**3**), β-rubromycin (**4**), rubromycin CA1 (**5**), or hyaluromycin (**6**) (Fig. 1). These rubromycin congeners are distinguished by alternative methylation patterns at the phenolic hydroxyl groups, different oxidation states of the A/B and/or spiroketal C/D rings, as well as the substitution patterns at the C1’ of the eastern isocoumarin moiety, which can be methyl or carboxy(methylester) groups for the griseorhodins and rubromycins, respectively (Fig. 1). Furthermore, two congeners, **3** and **6**, have been described, featuring distinct C6-linked sugar and C1’-linked 2-amino-3-hydroxycyclopent-2-enone (C_5_N) substituents, respectively.

Compound **6** was isolated in 2014 and the formation and attachment of the C_5_N unit via an amide bond to C1’ of a rubromycin CA1 backbone (**5**) was proposed to be catalyzed by a distinct set of enzymes encoded in the corresponding biosynthetic gene cluster (BGC) (Harunari et al., 2014). This involves a putative 5-aminolevulinate synthase (5-ALAS), an acyl-CoA ligase (ACL) as well as an amide bond synthetase (ABS), which, however, have not yet been biochemically investigated. Here, we aimed to discover novel rubromycin congeners by leveraging prior biosynthetic knowledge through genome-mining-guided identification of BGCs encoding unique tailoring enzymes, resulting in the discovery of coumarubrin (**7**), which features a distinct aminocoumarin connected via an amide bond to C1’ of a **5** backbone.

## Materials and Methods

### Materials

All chemicals and reagents used during this study were obtained from Sigma-Aldrich (St. Louis, MO), Alfa Aesar (Haverhill, MA), Carl Roth (Karlsruhe, Germany), and Fisher Scientific (Hampton, NH). The strain *Lentzea tibetensis* DSM No. 104 975 was obtained from the DSMZ strain collection (DSMZ-German Collection of Microorganisms and Cell Cultures GmbH, Braunschweig, Germany).

### Bioinformatic Tools

To search for genes homologous to the ABS from *S. hyaluromycini*, protein BLAST analysis of the amino acid sequence via the National Center for Biotechnology Information (NCBI) website was performed. Investigation of genomic DNA from potential rubromycin producers like *L. tibetensis*, was conducted via the antiSMASH 6.0 genome mining platform (Blin et al., 2021). For analysis and representation of BGCs, the CompArative GEne Cluster Analysis Toolbox (CAGECAT) with its clinker and cblaster modules were used (Gilchrist & Chooi, 2021; Gilchrist et al., 2021; van den Belt et al., 2023).

### Cultivation

For general handling, *L. tibetensis* was maintained on soy flour mannitol (20 g/l mannitol, 20 g/l soy flour, 20 g/l agar) and yeast-starch agar (10 g/l soluble starch, 2 g/l yeast extract, 15 g/l agar) plates. For compound production, a 50 ml R2A medium (5 g/l soluble starch, 5 g/l glucose, 2.5 g/l tryptone, 2.5 g/l yeast extract, 0.5 g/l casein [acid hydrolyzed], 0.25 magnesium sulfate heptahydrate, 0.5 g/l K_2_HPO_4_ adjusted to pH 7.0) seed culture in a 250 ml 3-baffled flask was cultivated for 5 days at 28°C and 130 rpm with an Infors shaker (Infors HT Multitron Triple Incubator Shaker). The main culture was performed with 800 ml mR2A medium in 2 l 3-baffled flasks inoculated with the seed culture at a ratio of 1% (v/v) and incubated at 28°C and 130 rpm for 7 days.

### Extraction

Harvested cells were diluted with water and lysed by sonication. Lysed cells were acidified to pH 5.5 and extracted extensively by vigorous stirring with the addition of three equivalent volumes of the organic extraction solvent mixture (4:1 EtOAc: THF). Organic solvent was evaporated under reduced pressure and crude extract was lyophilized before storage at −20°C until further use.

### Crude Extract Methylation

Crude extract methylation was performed as described before (Frensch et al., 2021). In short, to a crude extract (520 mg) suspension with K_2_CO_3_ (4.15 g) in anhydrous acetone (83.6 ml), dimethyl sulfate (8.32 g) was added dropwise. The reaction was stirred at room temperature for 1 hr and refluxed overnight. Afterwards, the reaction was cooled to room temperature and quenched by the addition of 1.5 volumes of water and further stirring for 1 hr. The whole mixture was extracted three times with equal volumes of EtOAc. The organic fractions were dried with anhydrous sodium sulfate and EtOAc was removed under reduced pressure.

### Compound Purification

Crude extract and methylated crude extract were first purified by silica purification on an Interchim Puriflash 4100 system. The material was adsorbed to celite for dry load application onto an Interchim PF-15SIHP-F0040 silica cartridge. The column was eluted with a four-solvent system containing hexane (A), DCM (B), EtOAc (C), and methanol (D) with a flow rate of 20 ml/min. The applied gradient was 100% A from 0 to 5 min, followed by a gradient to 100% B at 10 min. The DCM elution was held for 5 min, followed by a flat gradient to 10% C in B at 25 min, 10% C was held for 40 min followed by an increase to 25% over 10 min. Again, 25% C was held for 35 min before increasing to 40% C over 10 min and holding for 20 min. Finally, C was increased to 100% over 10 min and immediately to 100% D over 5 min which was held for another 5 min.

Further purification was performed on an Agilent 1100 HPLC equipped Phenomenex Kinetex 5 µM Biphenyl 100 Å LC column (250 × 10.0 mm). The applied gradient reached from 65% acetonitrile in water with 0.1% (v/v) formic acid for the first 2 min and a following gradient up to 80% acetonitrile with 0.1% (v/v) formic acid over 20 min. Each run was concluded by a 100% acetonitrile wash phase and following reequilibration.

### LC-MS Analysis

For standard measurements of purification fractions, liquid chromatography-mass spectrometry (LC-MS) measurements were performed on the Shimadzu LCMS-8030 Triple Quad Mass Spectrometer. Sample analysis was done on a SunFire C18 column (150 × 3 mm ID, 3.5 µm, Waters) equipped with a guard column (10 × 3 mm ID). Elution proceeded with 5% acetonitrile in water with 0.1% (v/v) formic acid for the first 2 min followed by a 20-min gradient up to 100% acetonitrile with 0.1% (v/v) formic acid. After that a column wash at 100% acetonitrile and subsequent equilibration to the starting condition for 5 min followed. UV/Vis absorption was monitored from 190 to 800 nm during the whole measurement. MS analysis in positive and negative mode proceeded with a capillary voltage of 3 kV, 250°C DL temperature, 400°C heat block temperature and 3 l/min nebulizing gas flow.

### LC-HRMS Measurements

For high resolution mass spectrometry an Agilent-UPLC system (Agilent Technologies, Santa Clara, CA) with an Agilent 1290 Binary pump G4220A, Agilent 1290 Infinity Autosampler G4226A, Agilent 1290 Infinity Thermostat G1130B, Agilent 1290 Thermostatted Column Compartment G1316C and Agilent 1290 Infinity Diode Array Detector G4212A connected to a Q-Exactive HF Orbitrap mass spectrometer (Thermo Scientific, Waltham, MA) was used. The HESI-II ion source was set to 3.5 kV with a sheath gas flow rate of 55 units, an auxiliary gas flow rate of 15 units, sweep gas flow rate of 0 units and an auxiliary gas temperature of 250°C. The ion transfer capillary temperature was set to 350°C and the RF-lens level to 45 units. Samples were separated with an ACQUITY UPLC BEH C18 column, 130 Å, 1.7 µm, 2.1 mm × 150 mm (Waters, Milford, MA), connected to a precolumn of the same phase. The applied method was a gradient elution. As mobile phase water and acetonitrile each supplemented with 0.1% formic acid were used. The method started with 5% acetonitrile for the first 0.5 min, followed by a gradient up to 100% acetonitrile until 15.5 min and a wash at 100% acetonitrile until 17.30 min, followed by reequilibration to 5% acetonitrile for 3 min. HRMS data were processed by MzMine 4.5.20 (Schmid et al., 2023) and analyzed with SIRIUS 6.1.0 software for calculation of the molecular formula and investigation of fragmentation patterns (Xing et al., 2023).

### NMR Measurements

The purified compounds were dissolved in 120 µl of deuterated DMSO and transferred into a 3 mm NMR tube. The spectra were recorded on a Bruker Avance III NMR spectrometer operating at 500.13 MHz and 125.77 MHz for ^1^H and ^13^C nuclei, respectively. ^1^H, ^13^C, HSQC, HMBC, and COSY spectra were recorded at 23°C on a BBO probe.

### Electronic Circular Dichroism Measurements

Electronic circular dichroism (ECD) spectra were recorded on a Chirascan Plus CD spectrometer (Applied Photophysics Ltd., Surrey, UK) equipped with a thermostatted cuvette holder. Measurements were performed at 25°C in a wavelength range from 195 to 400 nm (1 nm bandwidth, 3 s per data point). Samples were dissolved in HPLC-grade acetonitrile at a concentration of 0.2 mg/ml and placed in 1 mm path-length quartz cuvettes (Hellma GmbH, Müllheim, Germany). Data was recorded and analyzed with the Pro-Data V2.4 software (Applied Photophysics).

### Minimal Inhibitory Concentration

Minimal inhibitory concentrations (MICs) of coumarubrin (**7**) were determined according to the Clinical and Laboratory Standards Institute guidelines (Weinstein et al., 2018; Wikler, 2006) with some alterations. The following strains were obtained from the DSMZ: *Bacillus subtilis* (DSM 23778), *Micrococcus luteus* (DSM 20030), *Escherichia coli* (DSM 498), *Pseudomonas fluorescens* (DSM 50090), and *Saccharomyces cerevisiae* (DSM 1334). Precultures of bacterial strains were prepared by inoculating 25 ml sterile LB media in a 100 ml Erlenmeyer flask with cryostocks of the respective strains. The precultures were incubated in a shaker incubator overnight (*E. coli*: 37°C; *B. subtilis* and *M. luteus*: 35°C; *P. fluorescens*: 30°C). The optical density at 600 nm (OD_600_) was determined, and the cultures diluted to 5 × 10^5^ CFUs/ml in Cation-adjusted Mueller-Hinton Broth based on a previously established OD_600_–CFU relationship of overnight cultures. For *S. cerevisiae*, the cryostock was streaked out on Sabouraud dextrose agar containing 4% glucose and incubated for 24 hr at 35°C. Around 3–6 single colonies were transferred and resuspended in 2 ml sterile H_2_O. The OD_530_ was determined and the suspension diluted to 2 × 10^3^ CFUs/ml in Universal Yeast Medium (3 g/l yeast extract, 3 g/l malt extract, 5 g/l peptone from soybeans, 10 g/l glucose).

A twofold dilution series of **7** was prepared in DMSO. Two microliter of the dilution stocks or 2 µl of DMSO as a control were transferred to sterile 96-well clear flat-bottom microplates (Greiner, Art.-Nr. 655 161) and mixed with 198 µl of the diluted bacterial or fungal culture (four replicates) or sterile media as control. The highest final concentrations of **7** in the assays were 1 µM (*B. subtilis* and *M. luteus*) or 8 µM (*E. coli, P. fluorescens* and *S. cerevisiae*). The microplates were covered using a sterile breathable seal (Greiner, Art.-Nr. 676 051) and incubated on a microplate shaker (ELMI thermostatic shaker DTS-2) for 20 hr at 900 rpm at the corresponding temperatures (*E. coli*: 37°C; *B. subtilis* and *M. luteus*: 35°C; *P. fluorescens*: 30°C). The microplates containing *S. cerevisiae* were shaken for 10 min at 900 rpm and 35°C to ensure proper mixing of the compound and were then incubated for 24 hr at 35°C without agitation.

The MIC value was determined by identifying the lowest concentration of **7** at which no microbial growth could be observed compared to the media controls. For this, the ODs were measured (bacteria: 600 nm; yeast: 530 nm) using a microplate spectrophotometer (Thermo Scientific, Multiskan Go) and the wells were also inspected visually.

## Results and Discussion

**Fig. 2.**
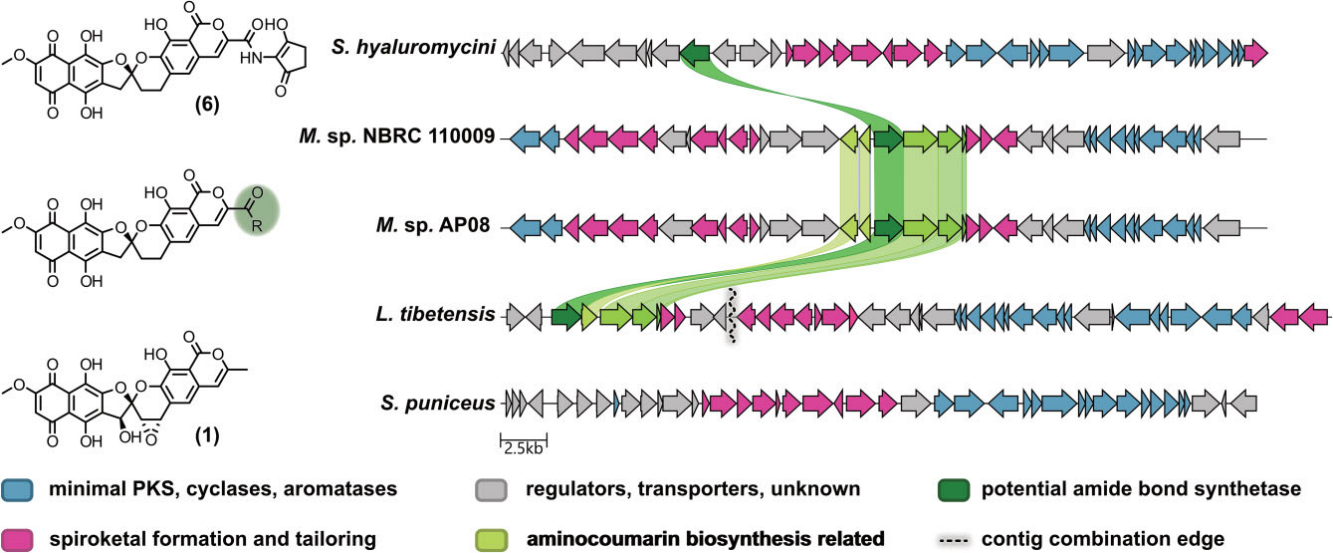
Representation of BCCs of *S. hyaluromicini* for production of **6**, *S. puniceus* for production of **1**, as well as *L. tibetensis, Micromonospora* sp. AP08 and *Micromonospora* sp. NBRC 110009 for the production of a novel rubromycin congener investigated in this work. Genes are represented as arrows and grouped by the (predicted) function of the encoded proteins, as stated in the color legend. Homologs coding for the ABS (dark green) and potential aminocoumarin biosynthesis enzymes (light green) are connected via colored ribbons. Further information regarding BGC composition, predicted function of the encoded proteins, and homology to characterized pathways can be found in Table S1 and Fig. S2. The whole genome sequencing data of *L. tibetensis, Micromonspora* sp. AP08, *Micromonospora* sp. NBRC 110009, *S. hyaluromicini* and *S. puniceus* can be found in the NCBI database and accessed via the identifiers ASM784567v1, ASM808593v1, ASM3051879v1, ASM221775v1 and ASM1690624v1, respectively.

Since the discovery of the first rubromycin polyketide, β-rubromycin (**4**), by Brockmann and Renneberg more than 70 years ago (Brockmann & Renneberg, 1953), numerous additional congeners have been isolated from different Actinobacteria. The intensively red-colored griseorhodins/rubromycins have interesting structural and functional properties but are typically poorly soluble and pose additional challenges during structural elucidation due to the many quaternary carbons and weak ^13^C-NMR signals. Recently, our group has studied late-stage enzymatic tailoring steps, in particular spiroketal biosynthesis involving several oxidases and oxygenases, allowing the functional assignment of numerous tailoring enzymes encoded in the various known rubromycin BGCs (Frensch et al., 2021; Toplak et al., 2022). As a result, previously uncharacterized rubromycin BGCs and corresponding compounds can be more easily predicted. We thus screened the genomic databases for putative novel rubromycin BGCs via the toolkit CAGECAT (van den Belt et al., 2023) combined with protein BLAST (Johnson et al., 2008). One aim was to find BGCs encoding putative ABSs similar to the hyaluromycin BGC that may attach alternative amines to the rubromycin backbone. In *Lentzea tibetensis* of the *Pseudonocardiaceae* family, a contig containing a partial BGC with 80% similarity to the β-rubromycin BGC was found, as also pointed out by the authors who first reported this strain (Huang & Huang, 2021). Upon closer inspection of the *L. tibetensis* genome, a separate contig was found with genes encoding putative rubromycin tailoring enzymes as well as a homolog of the hyaluromycin ABS (52.9% amino acid sequence identity). Interestingly, this contig also harbored additional genes encoding putative enzymes for aminocoumarin biosynthesis. Manual combination of the two contigs yielded the presumed complete rubromycin BGC of *L. tibetensis* with the genes coding for the ABS and aminocoumarin biosynthetic machinery bordering the core cluster (Fig. 2). In addition, highly similar rubromycin BGCs were found in two *Micromonospora* strains, in which the corresponding genes were rather integrated within the rubromycin BGC. Notably, the genes encoding 5-ALAS and ACL for C_5_N production were missing in all three strains, ruling out **6** biosynthesis.

The putative aminocoumarin biosynthesis genes identified in these BGCs encode homologs of proteins from novobiocin biosynthesis, that is NovH, NovI, an MbtH-like protein, as well as NovJ/K. The biosynthesis of the aminocoumarin motive of novobiocin is initiated by the non-ribosomal peptide synthetase (NRPS)-like enzyme NovL, which activates a tyrosine precursor supported by the auxillary MbtH-like protein, before the P450-enzyme NovI catalyzes beta-hydroxylation. Subsequently, the introduced hydroxyl group is oxidized by NovJ/K, whereas the following potential steps of aromatic ring hydroxylation as well as aminocoumarin ring closure have not been elucidated so far (Boll et al., 2011; Heide, 2009; Pacholec et al., 2005). These predicted functionalities suggested an aminocoumarin or a related amine as terminal substituent of the novel congener.

**Fig. 3.**
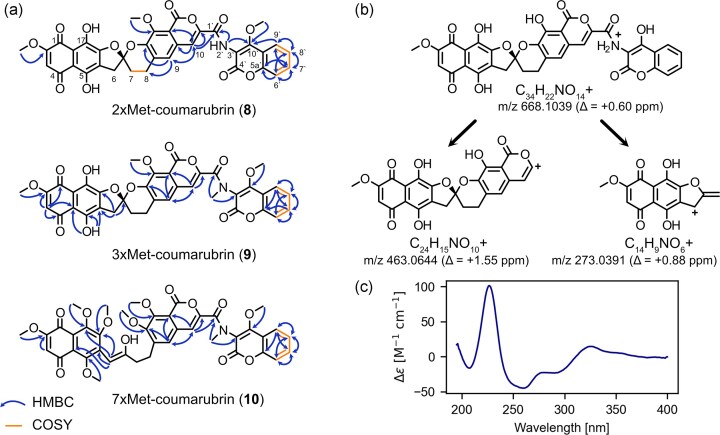
(A) Representation of the structures of the isolated compounds **8, 9, 10** as well as their most important HMBC (blue arrows) and COSY (orange bars) correlations. Representative atom numbering is shown for compound **8**. (B) Proposed structures of the most dominant ions and observed fragments in HRMS/MS experiments. (C) Experimental ECD spectrum recorded for 2xMet-coumarubrin (**8**).

To further investigate this, the strain *Lentzea tibetensis* DSM No. 104 975 was obtained from the DSMZ-German Collection of Microorganisms and Cell Cultures GmbH and cultivated under several different conditions on agar plates and in liquid media to investigate for secondary metabolite production. Following the optimization of media and cultivation conditions, the bacteria eventually showed bright purple-pink coloration of the cell material and to a lesser extent in the culture supernatant. Analysis of the UV/Vis-spectrum showed characteristic rubromycin absorption features and high-resolution MS/MS experiments revealed a molecular formula of C_34_H_22_NO_14_^+^ ([M + H]^+^ adduct, measured: 668.1040, calculated: 668.1035) and characteristic fragments of a rubromycin scaffold (see Fig. 3B and Figs S3–S5). Large-scale extraction and purification yielded a compound with extremely poor solubility in all commonly used (NMR) solvents, initially preventing further structure elucidation (∼1.0 mg of pure compound per liter of culture). However, in one of the purification attempts, an acid degradation product could be obtained and analyzed via NMR with matching chemical shifts to the known rubromycin CA1 (**5**), which features a non-oxygenated spiroketal moiety (see Fig. S6, Table S2 and Figs S7 and S8) (extraction with ethyl acetate or methanol containing 0.1% [v/v] trifluoroacetic acid caused substantial degradation). To confirm the identity of the terminal ligand attached to the rubromycin core, the crude extract of *L. tibetensis* was subjected to chemical methylation using dimethyl sulfate (DMS), which was shown before to increase solubility and facilitate purification and structural elucidation of griseorhodins/rubromycins (Frensch et al., 2021; Harunari et al., 2014). At first, partially and later also fully methylated species of this rubromycin congener could be obtained, of which compounds **8, 9**, and **10** were then successfully purified and analyzed via NMR (see Fig. 3A).

The NMR spectra and HRMS/MS analysis of all isolated compounds are in agreement with a rubromycin featuring a 3-amino-4-hydroxy-coumarin connected to C1’ via an amide bond (see Figs S9–S33 and Table S3). The general structure of the rubromycin backbone was already indicated from the isolation of rubromycin CA1 and the HRMS results were confirmed by the NMR spectra of **8, 9**, and **10**. HMBC correlations to the quarternary carbons of the naphthoquinone moiety of ring A and B were observed for compounds **9** and **10**. The non-oxygenated spiroketal moiety was confirmed by the CH_2_ signals of positions C6, C7, and C8. The hydrolysed spiroketal of compound **10** resulted in a keto-enol tautomerism for C6 and C6a also visible in the NMR spectra (here only the enol form is shown). Characteristic signals for protons and respective HMBC and COSY correlations at position C9 and C10 of the isocoumarin were detected for all compounds. For **8** and **10**, an HMBC correlation of position C10 to C1’ confirmed the amide bond linkage. In addition, HMBC correlations to C1’ were seen for the amide proton of **8**, as well as for the respective methyl groups of **9** and **10**. For **8**, the amide proton shows an HMBC signal to C10’ whereas the amide methyl groups of **9** and **10** show HMBC correlations to C3’, verifying the C1’-connectivity of the aminocoumarin moiety. The aromatic protons of C6’, C7’, C8’, and C9’ show HMBC correlations to each other and C5a’ as well as to C9’–C10’ in case of **8**. Additionally, all protons of C6’, C7’, C8’, and C9’ are connected by COSY correlations. Likely due to its far distance, no HMBC correlation to position C4’ could be observed. Still, based on the chemical shift of the isolated compounds, the lack of any methylated product from a hypothetical carboxylic acid and the HRMS results with matching molecular formulas for all the structures, the possibility of a ring-opened, hydrolyzed lactone of the aminocoumarin moiety could be ruled out. Notably, a lack of HMBC correlations to the lactone carbonyl group was also reported for other aminocoumarin natural products (Sasaki et al., 2001). It is noteworthy that the hydroxyl group at the C7’ position of the aminocoumarin moiety of **7** is missing in contrast to other aminocoumarin antibiotics (e.g. novobiocin, coumermycin and clorobiocin (Heide, 2009)), suggesting that the biosynthesis involves a phenylalanine precursor instead of tyrosine (Fig. 1). Indeed, closer inspection of the predicted A-domain specificity of the NRPS-like NovH homolog of the *L. tibetensis* BGC corroborated a phenylalanine as a likely starter unit (see Fig. S34).

To investigate the configuration of the spiroketal moiety of **7**, ECD measurements of the dimethylated derivative **8** were performed. The obtained spectrum featured a positive cotton effect around 225 nm and a negative cotton effect at 260 nm (see Fig. 3C). These characteristic spectral properties were also found for hyaluromycin (**6**) (Harunari et al., 2014), rubromycins CA1 (**5**) and CA2 (Harunari et al., 2019), β-rubromycin (**4**), γ-rubromycin and griseorhodin A (**1**) (Bringmann et al., 2000; Yunt et al., 2009), suggesting the same spatial arrangement and configuration of the spiroketal moieties of these griseorhodins and rubromycins. It is noteworthy that in case of griseorhodins A (**1**) and C, the proposed spiroketal stereochemistry was recently reassigned from *S* to *R* due to a prior misapplication of the Cahn-Ingold-Prelog (CIP) priority rules (Ortega et al., 2017). Despite the *S*/*R* label difference, **1** shares the same spiroketal spatial orientation as the other rubromycins, with the stereochemical discrepancy arising from differing CIP priority rules caused by variable substitution patterns on the spiroketal. Interestingly, heliquinomycin (**3**), the only rubromycin crystallized to date, exhibits an *R*-configured spiroketal moiety with a spatial arrangement of the spiroketal moiety distinct from that of other griseorhodins and rubromycins (Chino et al., 1997). This is also supported by experimental CD spectra showing nearly mirrored curves and opposite cotton effects for **3** compared to γ-rubromycin (Bringmann et al., 2000). In light of our previous results, we thus propose an *S*-configuration of coumarubrin (**7**), as depicted in Fig. 1.

Typically, rubromycins show potent antibiotic activity against Gram-positive strains. However, **7** contains an unusual and sizeable substituent compared to other rubromycins, and we thus tested its antimicrobial activity by determining the minimal inhibitory concentration (MIC) for several bacteria and the yeast *Saccharomyces cerevisiae*. The results show potent activity against Gram-positive strains *B. subtilis* and *M. luteus* but no inhibition of baker's yeast or the Gram-negative *E. coli* and *P. fluorescens* at the tested concentrations (see Table 1). Overall, this antimicrobial activity profile appears comparable to other rubromycins (Atkinson & Brimble, 2015).

**Table 1.** Minimal Inhibitory Concentration (MIC) Values of Coumarubrin (**7**) Against Gram-Positive (*B. subtilis* and *M. luteus*), Gram-Negative (*E. coli* and *P. fluorescens*) and Yeast (S*. cerevisiae*) Strains

Organism	MIC value in µM (µg/ml)
*B. subtilis*	0.25 (0.167)
*M. luteus*	0.25 (0.167)
*E. coli* K12	>8 (>5.340)
*P. fluorescens*	>8 (>5.340)
*S. cerevisiae*	>8 (>5.340)

All conditions were tested based on the guidelines of the Clinical and Laboratory Standards Institute (see Materials and Methods).

It is noteworthy that the aminocoumarin moiety of the molecule could possibly hint at an inhibitory activity against bacterial DNA gyrase, as this is a common target of other aminocoumarin antibiotics (Heide, 2009). Moreover, rubromycins are well-known to interfere with enzymes related to DNA/RNA processes (Atkinson & Brimble, 2015). Further detailed investigation of the bioactivities and potential mode of action of **7** are thus warranted.

## Conclusion

Taken together, following bioinformatic prediction, the new rubromycin congener coumarubrin (**7**) was identified in this study featuring a 3-amino-4-hydroxy-coumarin substituent. The identified BGC of the producer strain contained not only rubromycin biosynthesis genes but also genes encoding enzymes for aminocoumarin biosynthesis as well as an ABS, which likely connects both scaffolds. The aminocoumarin-fused **7** thus expands the structural diversity of the rubromycin type family and its discovery showcases how genomics can be leveraged to unearth novel rubromycins more than 70 years after the discovery of the first congener.

## Supplementary Material

kuaf018_Supplemental_File

## Data Availability

The data not included within the article or the supplementary information will be shared on reasonable request to the corresponding author.
