# Supplementary material for: Genome-mining-guided discovery of coumarubrin: A novel aminocoumarin-substituted rubromycin antibiotic
Source: J Ind Microbiol Biotechnol. 2025 Jul 4;52:kuaf018. doi: 10.1093/jimb/kuaf018 (PMC12284476; doi:10.1093/jimb/kuaf018)
Supplement: kuaf018_Supplemental_File [file kuaf018_supplemental_file.docx]

**Supporting information**

# **Genome-mining-guided discovery of coumarubrin: A novel aminocoumarin-substituted rubromycin antibiotic**

Heiner G. Weddeling^1^, Sven T. Sowa^1^, Selina Bosshardt^1^, Lukas Schwimbersky^1^, Malik Rakhmanov^1^, Robin Teufel^1*^

[1] Pharmaceutical Biology, Department of Pharmaceutical Sciences, University of Basel, Klingelbergstrasse 50, 4056 Basel (Switzerland)

^*^E-mail: [robin.teufel@unibas.ch](mailto:robin.teufel@unibas.ch)

**Figure S1**: Representation of the proposed simplified coumarubrin (**7**) biosynthetic pathway including enzymatic steps specific to rubromycin biosynthesis, i.e. oxidation of the isocoumarin methyl group of the griseorhodins to the corresponding carboxylic acid found in rubromycins (Frensch et al., 2021; Toplak et al., 2022; Yi et al., 2024). In **3** biosynthesis, this carboxylic acid is likely converted to an amide by the putative amide bond synthetase (LtiABS) that attaches the aminocoumarin substituent.


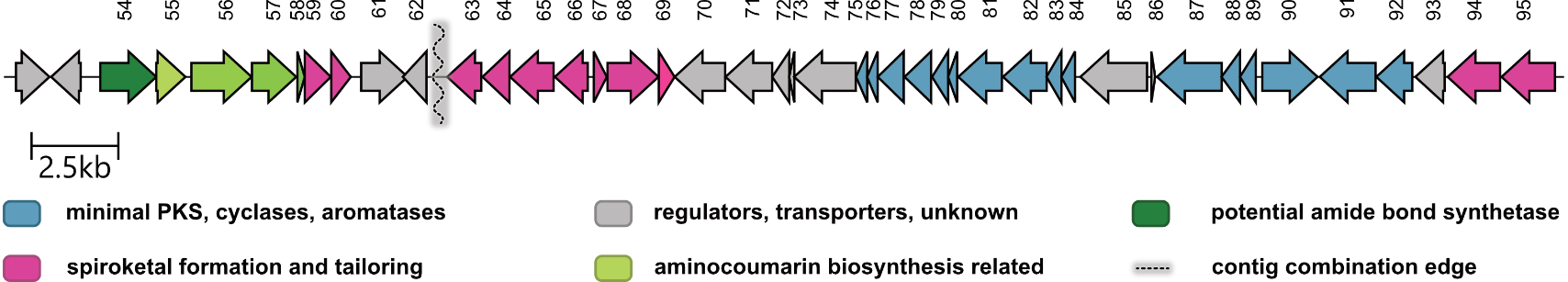


**Figure S2**: Representation of the biosynthetic gene cluster of *L. tibetensis* with the color-coded hypothetical gene function. All genes have been numbered according to their contig number of the genome sequence (compare to table S1).

**Table S1:** ORFs of the coumarubrin BGC with the predicted function of the encoded proteins in comparison to the *rub* and *grh* BGCs. “-“ indicated the absence of homologous genes/proteins. The genes of *the L. tibetensis* BGC are numbered according to the combined contigs containing the whole cluster (compare to figure S2). For all genes and their protein products the NCBI identifier is given.

| ***cou* BGC (*L. tibetensis*)** | **hypothetical/annotated function** | ***rub* BGC (*S. collinus*)** | ***grh* BGC (*S.* sp. JP95)** |
| --- | --- | --- | --- |
| - | efflux pump | - | GrhK, (AAM33663.1) |
| - | SAM-dependent methyltransferase | Orf2, (AAM97375.1) | - |
| 54 (WP_222598544) | AMP-binding protein (hypothetical amide bond synthetase) | - | - |
| 55 (WP_186763421.1) | ester cyclase | RubK, (AAM97361.1) | - |
| 56 (WP_222598543.1) | AMP-binding protein (novH) | - | - |
| 57 (WP_146359513.1) | P450 (novI homolog) | - | - |
| 58 (WP_146359510.1) | mbth-like protein | - | - |
| 59 (WP_146359507.1) | short-chain dehydrogenase (novJ homolog) | - | - |
| 60 (WP_146359504.1) | short-chain dehydrogenase (novK homolog) | - | - |
| 61 (WP_186763420.1) | drug resistance transporter | - | - |
| 62 (TWP52749.1) | unknown | - | - |
| 63 (WP_146353542.1) | NADPH: quinone oxidoreductase | - | GrhO7, (AAM33674.1) |
| 64 (WP_146353543.1) | polyketide ketoreductase | RubJ, (AAM97360.1) | GrhO2, (AAM33669.1) |
| 65 (WP_146353544.1) | cytochrome P450 monooxygenase | RubU, (AAM97370.1) | GrhO3, (AAM33670.1) |
| 66 (WP_186763054.1) | GNAT family N-acetyltransferase | - | GrhJ, (AAM33662.1) |
| 67 (WP_146353546.1) | unknown | - | GrhI, (AF509565) |
| 68 (WP_146353547.1) | FAD dependent oxidoreductase | RubI, (AAM97372.1) | GrhO1, (AIE76922.1) |
| 69 (WP_146353548.1) | transcriptional regulator | RubM, -(AAM97363.1) | GrhR3, (AAM33681.1) |
| 70 (WP_146353549.1) | major facilitator transporter | (AAM97376.1) | - |
| 71 (WP_146353550.1) | acetyl-CoA carboxylase | - | - |
| 72 (WP_146353551.1) | unknown (acetyl-CoA carboxylase) | - | - |
| 73 (WP_186763055.1) | unknown (acetyl-CoA carboxylase) | - | - |
| 74 (WP_146353751.1) | acetyl-CoA carboxylase | - | - |
| 75 (WP_146353552.1) | Antibiotic biosynthesis monooxygenase | RubT, (AAM97374.1) | GrhV, (AAM33684.1) |
| 76 (WP_146353553.1) | hypthetical protein | RubH, (AAG03072.1) | GrhU, (AAM33683.1) |
| 77 (WP_146353554.1) | SDR family NAD(P)-dependent oxidoreductase | RubG, (AAG03071.1) | GrhO10, (AAM33668.1) |
| 78 (WP_146353555.1) | polyketide cyclase/reductase | RubF, (AAG03070.2) | GrhT, (AAM33685.1) |
| 80 (WP_146353556.1) | acyl carrier protein | RubC, (AAG03069.1) | GrhC, (AAM33655.1) |
| 81 (WP_146353557.1) | ketosynthase beta subunit | RubB, (AAG03068.1) | GrhB, (AAM33654.1) |
| 82 (WP_146353558.1) | ketosynthase alpha subunit | RubA, (AAG03067.1) | GrhA, (AIE76940.1) |
| 83 (WP_146353559.1) | cyclase | RubD, (AAG03066.1) | GrhS, (AAM33682.1) |
| 84 (WP_328593149.1) | cyclase | RubE, (AAG03065.2) | GrhQ, (AAM33678.1) |
| 85 (WP_146353561.1) | transcriptional activator | RubS, (AAM97369.1) | GrhR2, (AAM33680.1) |
| 86 (no protein annotated) | unknown | - | - |
| 87 (WP_146353562.1) | asparagine synthase | RubR, (AAM97368.1) | GrhP, (AAM33677.1) |
| 88 (WP_146353563.1) | unknown | RubW, (AAM97367.1) | GrhN, (AAM33666.1) |
| 89 (WP_407936534.1) | unknown | RubQ, (AAM97373.1) | GrhM, (AAM33665.1) |
| 90 (WP_146353565.1) | FAD-dependent monooxygenase | RubP, (AAM97366.1) | GrhO9, (AAM33676.1) |
| 91 (WP_146353566.1) | FAD-dependent monooxygenase | - | GrhO8, (AAM33675.1) |
| 92 (WP_146353567.1) | methyltransferase | - | GrhL, (AAM33664.1) |
| 93 (WP_146353568.1) | SDR family oxidoreductase | - | - |
| 94 (WP_146353569.1) | polyketide oxygenase | RubN, (AAM97364.1) | GrhO6, (AAM33673.1) |
| 95 (WP_146353570.1) | polyketide hydroxylase | RubL, (AAM97362.1) | GrhO5, (AAM33672.1) |


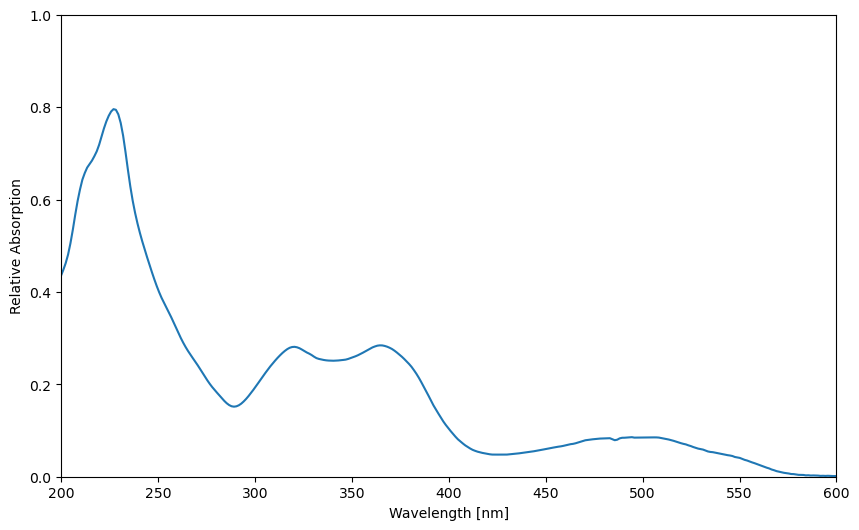


**Figure S3**: UV spectrum of coumarubrin featuring hallmark absorption characteristics of rubromycin type compounds with a broad absorption peak around 500 nm, a double absorption peak around 320 and 356 nm as well as highest absorption around 227 nm.


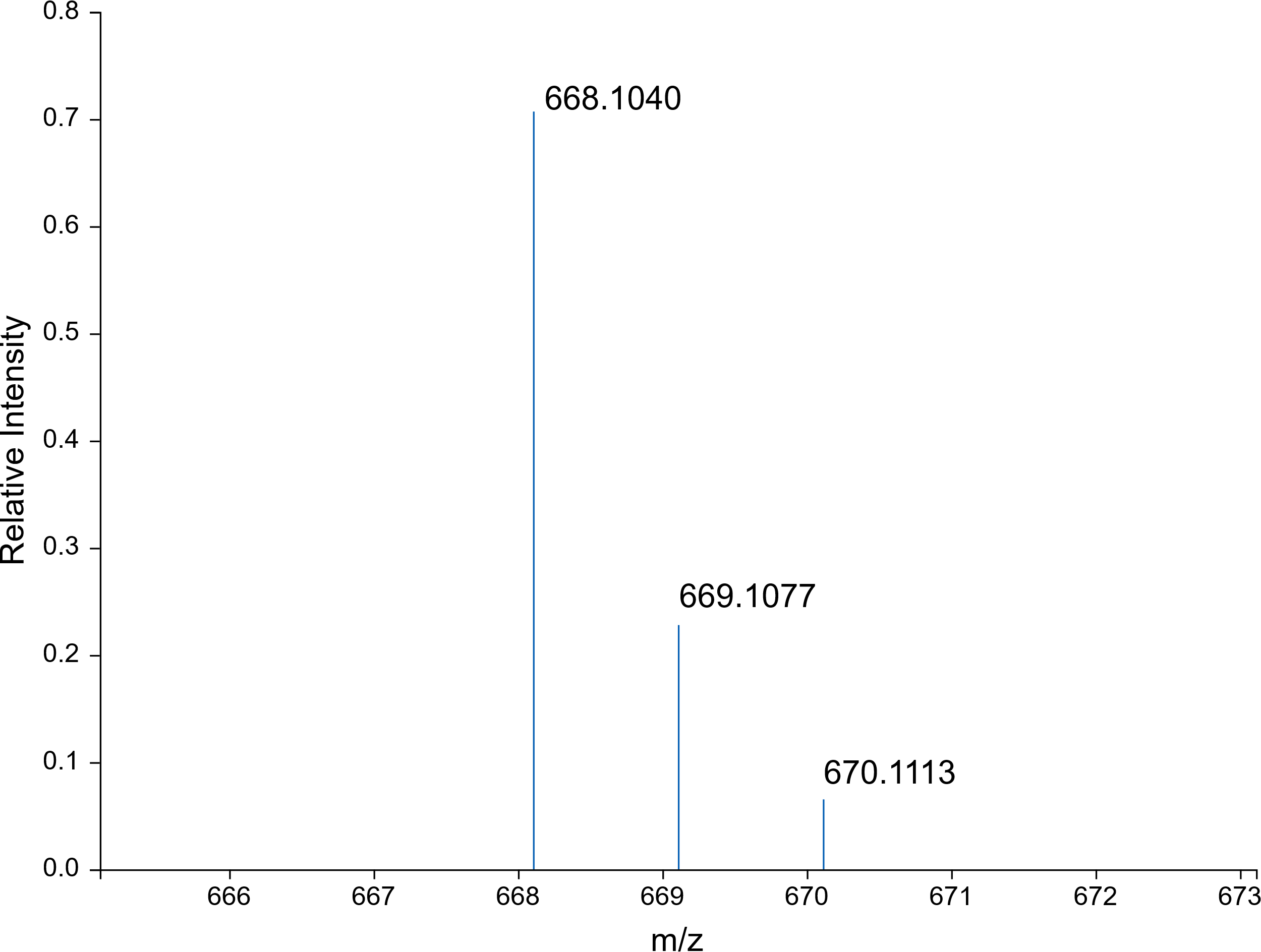


**Figure S4**: mass spectrum of coumarubrin (**7**) (C_34_H_21_NO_14_) measured in positive mode with the isotope pattern for the [M+H]^+^ adduct (measured: 668.1040, calc.: 668.1035).


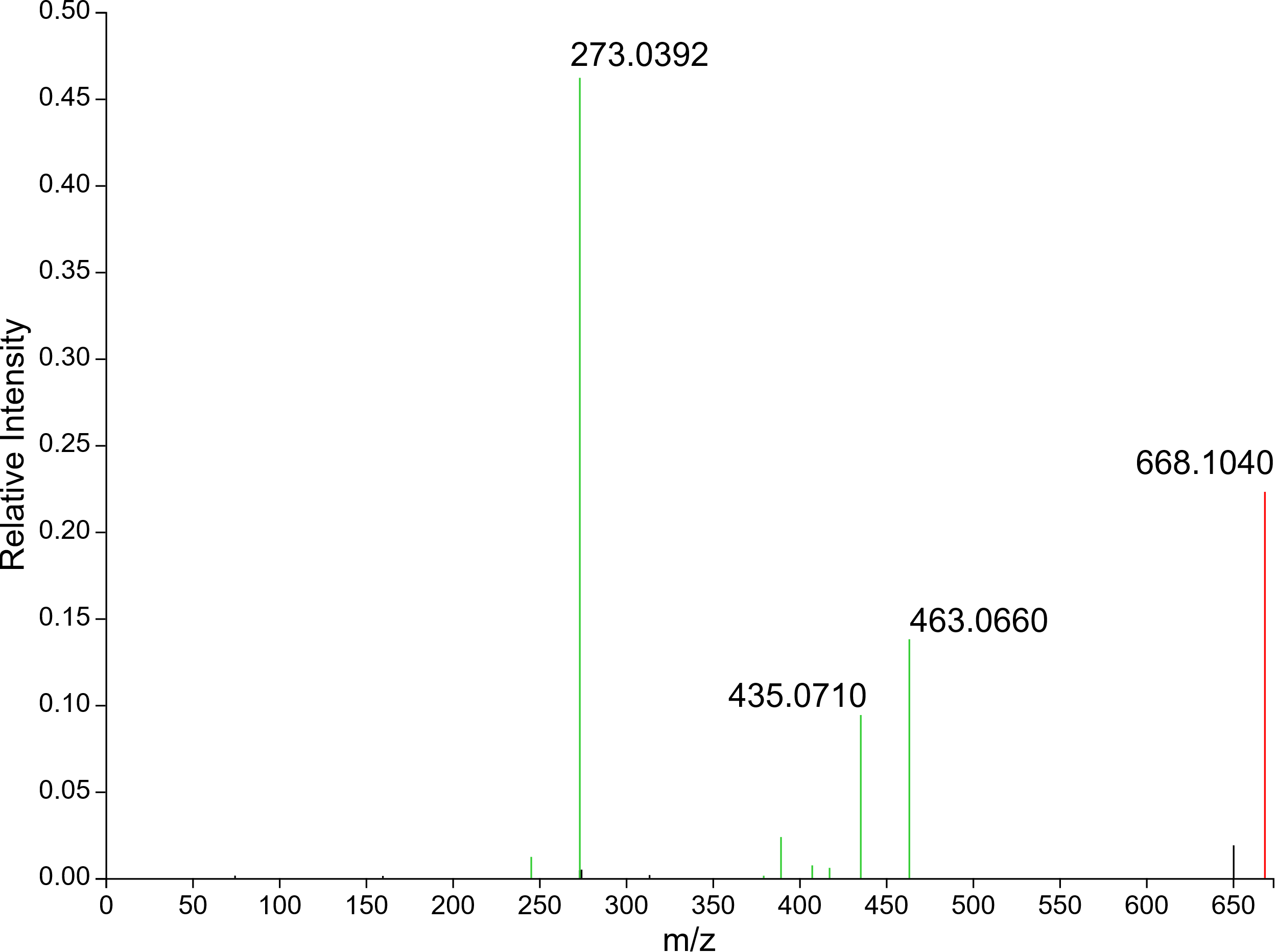


**Figure S5**: MS2 spectrum of coumarubrin (**7**) measured in positive mode, the three most prominent fragments are highlighted.

**Figure S6**: Representation of rubromycin CA1 that has been purified from a purification attempt with harsh acidification and analyzed by NMR and HRMS/MS in this study. Numbers for carbon atoms are given for each of the molecules. Since the rubromycin atom numbering is inconsistent in the literature, the second alternative atom numbering is also shown within the table of the NMR data of the following material.

**Table S2**: NMR ^1^H and ^13^C chemical shifts of Rubromycin CA1 isolated from *L. tibetensis* and the literature values measured in DMSO-d_6_. Atom numbering in the first column is shown as in Figure S4. and the alternative numbering (alt. No.) in the second column.

| atom | alt. No. | $\delta_{C}$^(a)^ | $\delta_{C}$^(b)^ | $\delta_{H}$^(a)^ | $\delta_{H}$^(b)^ |
| --- | --- | --- | --- | --- | --- |
| 2 | 7’ | 160.21 | 160.3 |  |  |
| 3 | 6’ | 109.64 | 110 | 6.40 | 6.4 |
| 6 | 3’ | 38.44 | 38.5 | 3.47 | 3.61 |
| 6 | 3’ | 38.44 | 38.5 | 3.61 | 3.47 |
| 7 | 3 | 28.24 | 28.2 | 2.36 | 2.57 |
| 7 | 3 | 28.24 | 28.2 | 2.56 | 2.36 |
| 8 | 4 | 21.73 | 21.7 | 3.16 | 3.15 |
| 8 | 4 |  |  | 3.05 | 3.05 |
| 8a | 4a | 131.68 | 131.7 |  |  |
| 9 | 5 | 118.35 | 118.7 | 7.21 | 7.25 |
| 9a | 5a | 127.99 | 127.7 |  |  |
| 10 | 6 | 111.77 | 112.1 | 7.59 | 7.61 |
| 13a | 9a | 106.24 | 106.4 |  |  |
| 14 | 10 | 148.97 | 149 |  |  |
| 14a | 10a | 140.00 | 140.4 |  |  |
| 1’ | 11 | 161.08 | 161 |  |  |
| 1’’ | 10’ | 57.03 | 57.1 | 3.89 | 3.89 |
| 5-OH | 4’-OH |  |  | 10.79 |  |
| 17-OH | 9’-OH |  |  | 13.15 | 13.13 |
| 14-OH | 10-OH |  |  | 11.90 | 11.87 |

(a) Recorded at 125 MHz for ^13^C and 500 MHz for ^1^H respectively (b) chemical shifts reported in (Harunari et al., 2019)


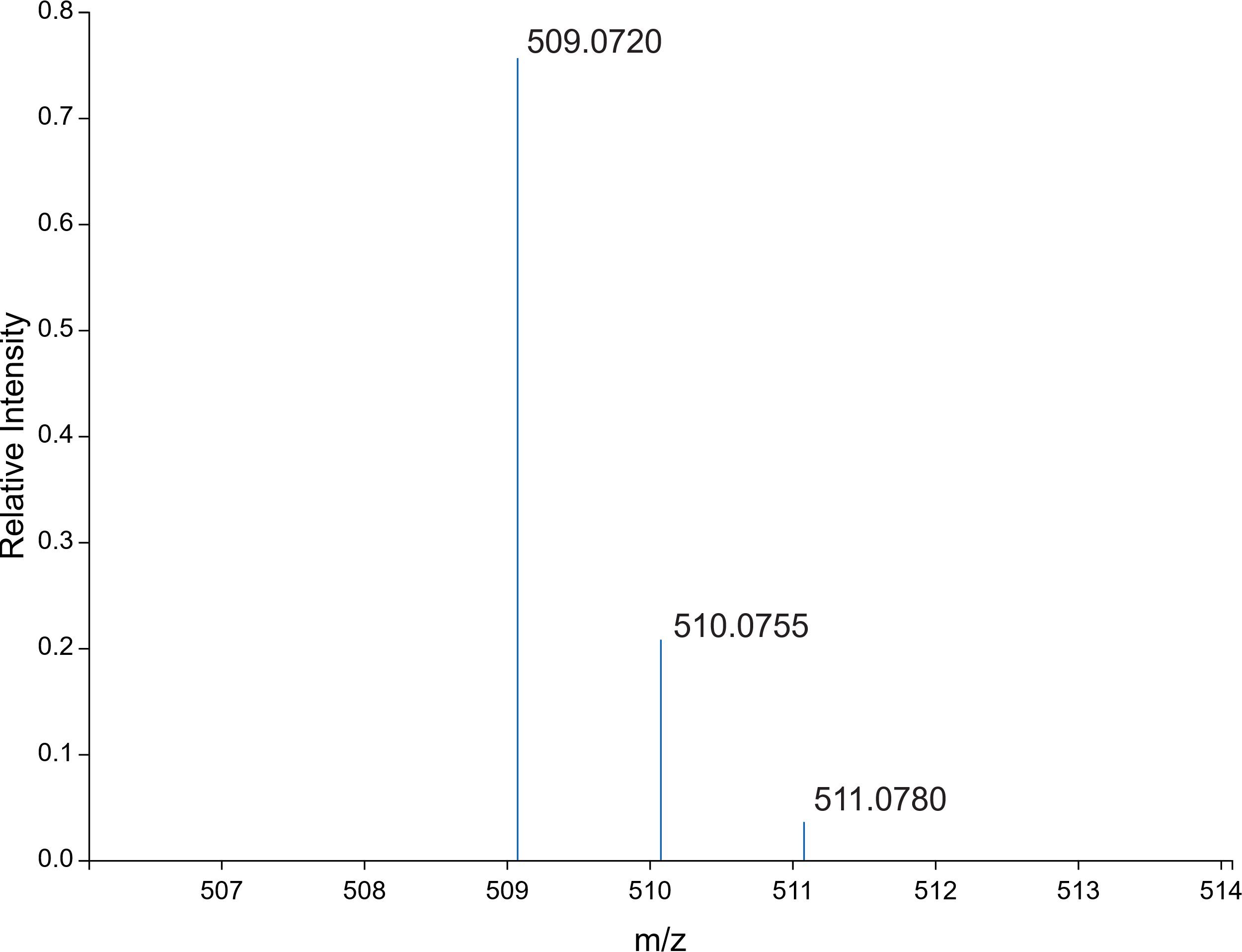


**Figure S7**: mass spectrum of rubromycin CA1 (**5**) (C_25_H_16_O_12_) measured in positive mode (measured: 509.0720, calc.: 509.0715).


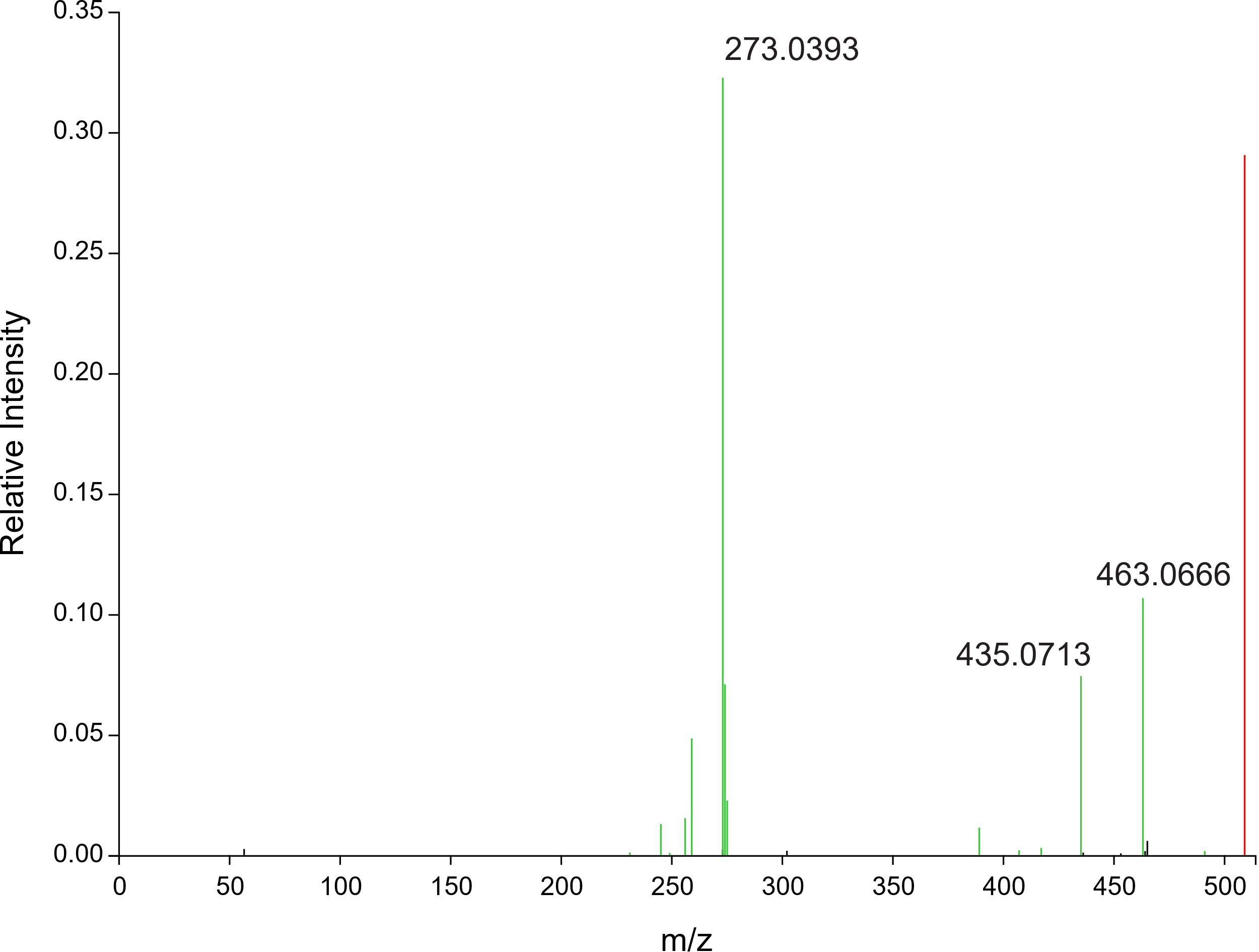


**Figure S8**: MS2 spectrum of rubromycin CA1 (**5**) measured in positive mode, the three most prominent fragments are highlighted.
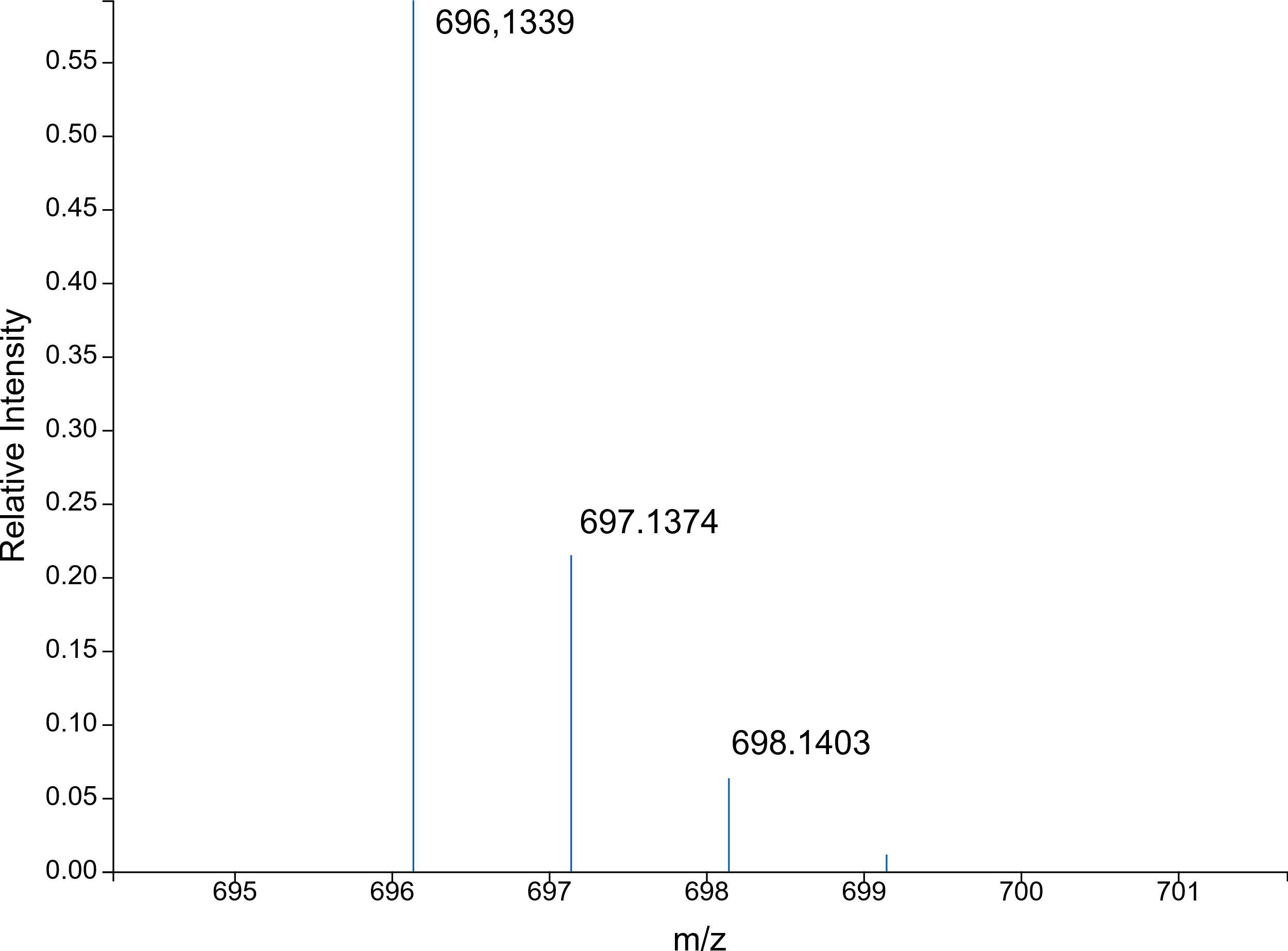


**Figure S9**: mass spectrum of 2xMet-coumarubrin (**8**) (C_36_H_25_NO_14_) measured in positive mode (measured: 696. 1339 calc.: 696.1348).


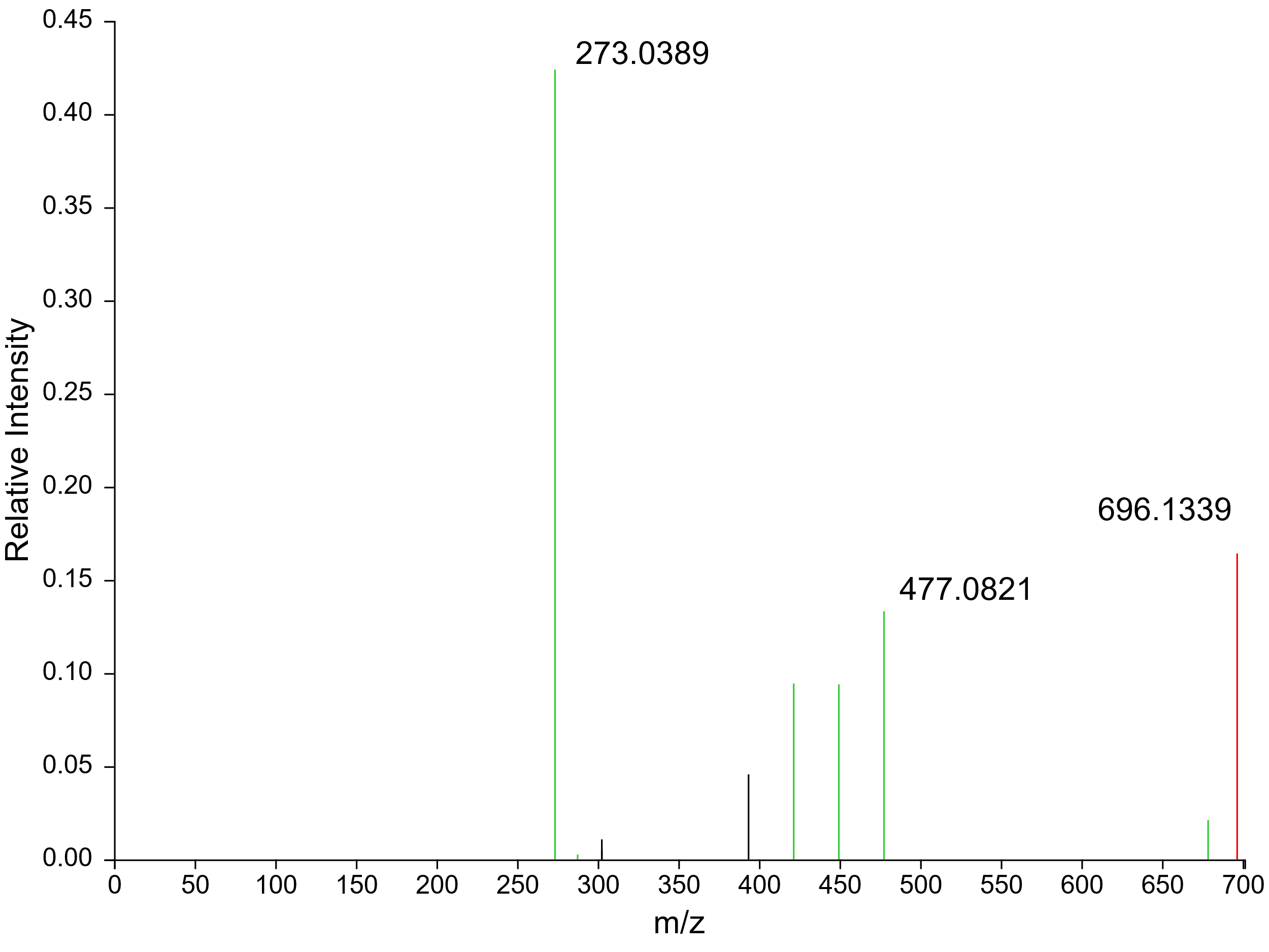


**Figure S10**: MS2 spectrum of 2xMet-coumarubrin (**8**) measured in positive mode, the two most prominent fragments are highlighted.


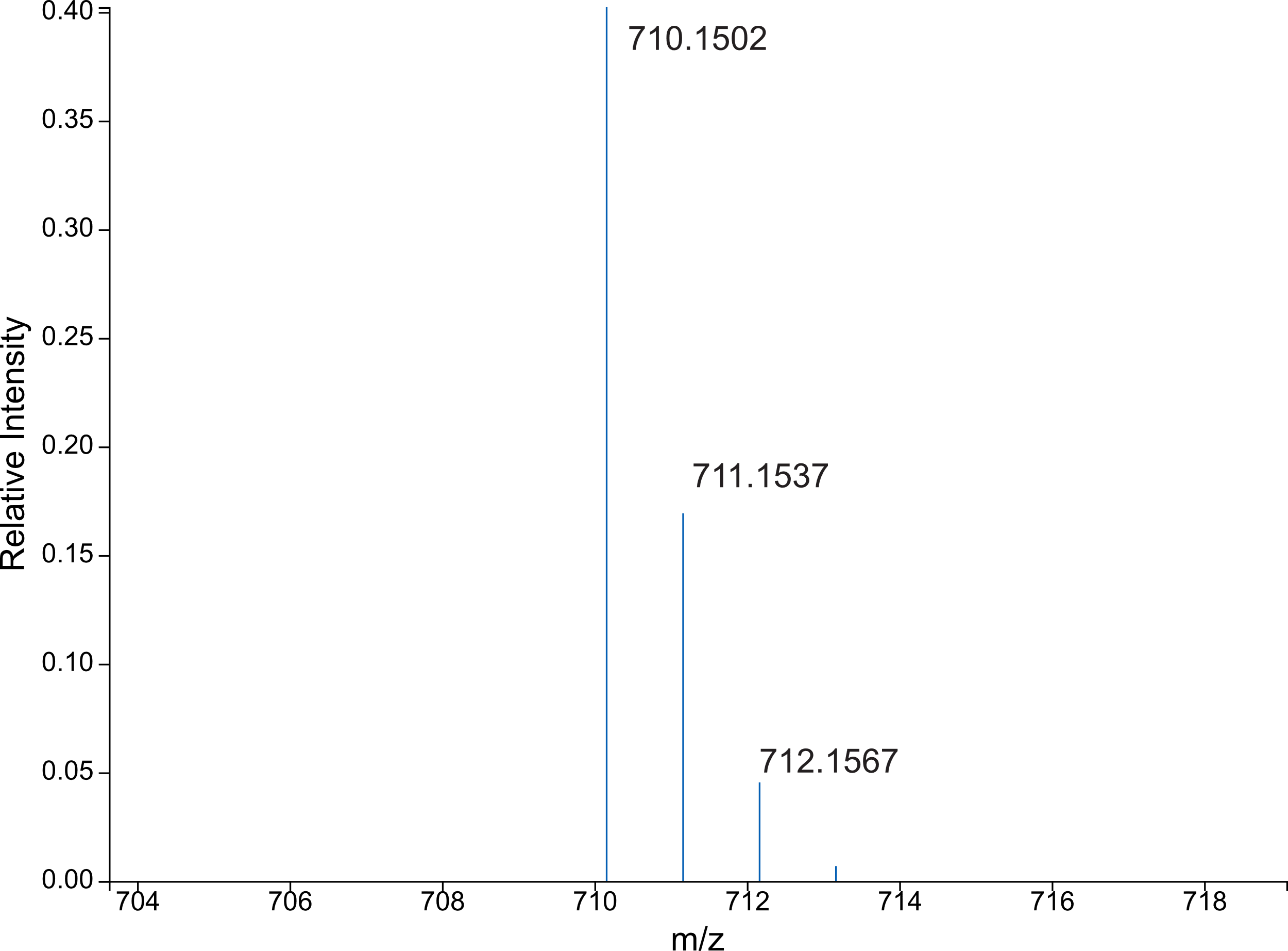


**Figure S11**: mass spectrum of 3xMet-coumarubrin (**9**) (C_37_H_27_NO_14_) measured in positive mode (measured: 710.1502, calc.:710.1504).


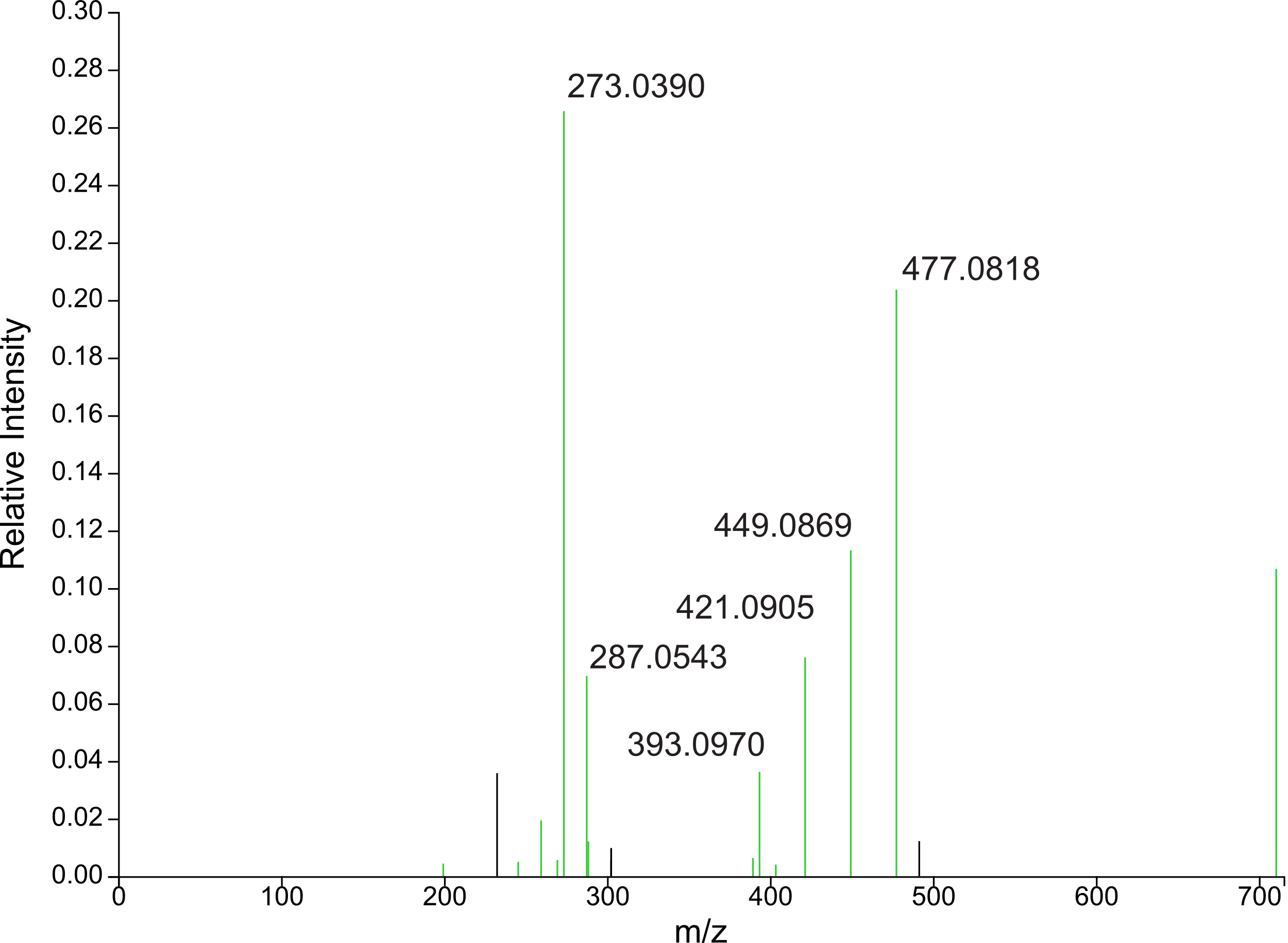


**Figure S12**: MS2 spectrum of 3xMet-coumarubrin (**9**) measured in positive mode, the six most prominent fragments are highlighted.


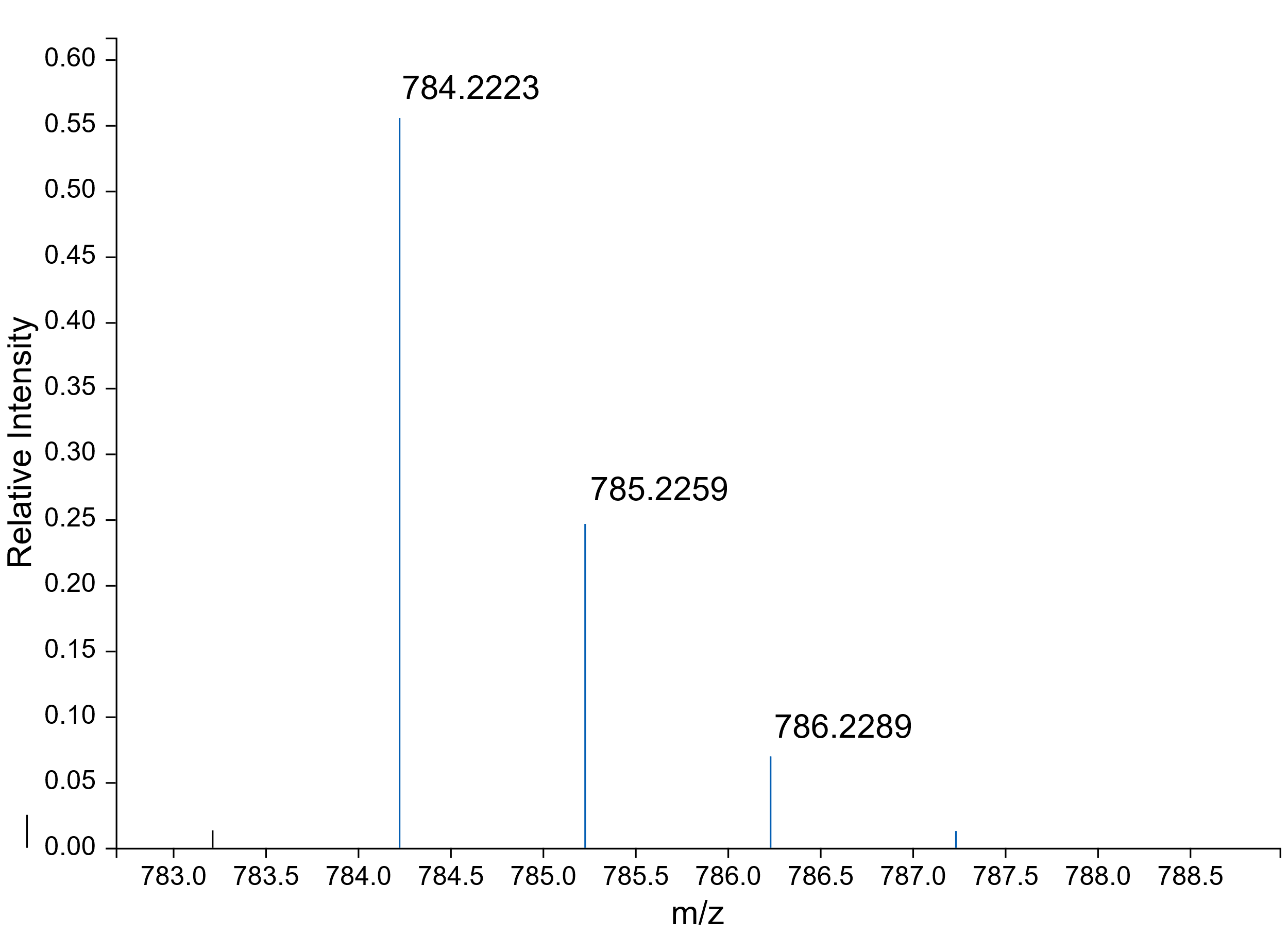


**Figure S13**: mass spectrum of 7xMet-coumarubrin (**10**) (C_41_H_37_NO_15_) measured in positive mode (measured: 784.2223 calc.:784.2236).


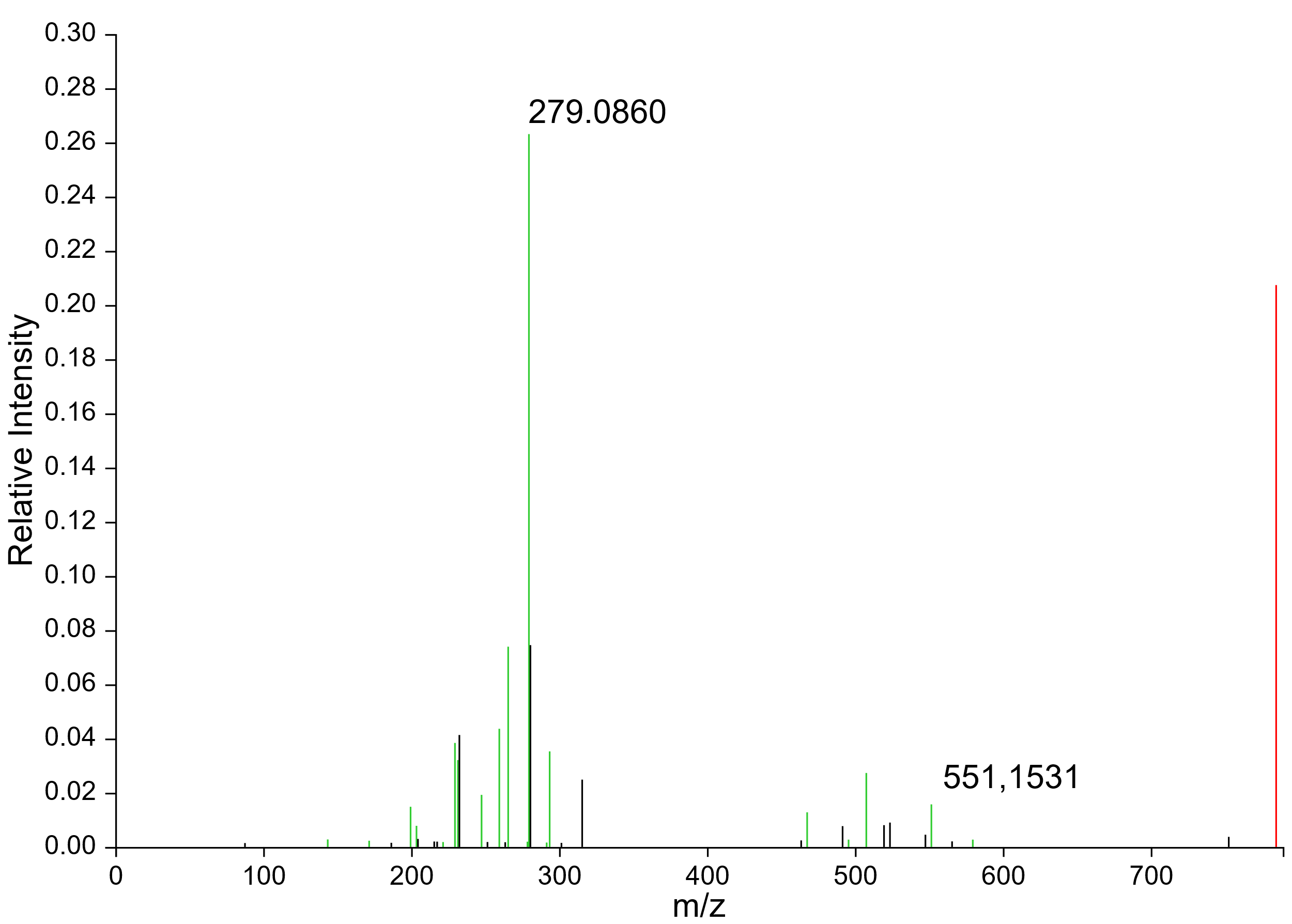


**Figure S14**: MS2 spectrum of 7xMet-coumarubrin (**10**) measured in positive mode, the two most prominent fragments are highlighted.

**Figure S15**: Representation of the three methylated coumarubrin derivatives 2xMet-coumarubrin (**8**), 3xMet-coumarubrin (**9**) and 7xMet-coumarubrin (**10**) that have been purified and analyzed by NMR and HRMS/MS in this study. Numbers for carbon atoms are given for each of the molecules. Since the rubromycin atom numbering is inconsistent in the literature, an alternative atom numbering is also shown in the tables containing the NMR data.

**Table S3**: NMR ^1^H and ^13^C chemical shifts of the three methylated coumarubrin derivatives **8**, **9** and **10** in DMSO-d_6_ (for compound **10** only the shifts for the enol form of the compound are noted). Atom numbering is given as in Figure S14. with the alternative numbering (alt. No.) shown in the second column.

|  |  | 2xMet-Coumarubrin (**8**) | | 3xMet-Coumarubrin (**9**) | | 7xMet-Coumarubrin (**10**) | |
| --- | --- | --- | --- | --- | --- | --- | --- |
| atom | alt. No. | $\delta_{C}$^(a)^ | $\delta_{H}$^(b)^, mult. | $\delta_{C}$^(a)^ | $\delta_{H}$^(b)^, mult. | $\delta_{C}$^(a)^ | $\delta_{H}$^(b)^, mult. |
| 1 | 8’ |  |  | 179.5 |  |  |  |
| 2 | 7’ | 160.4 |  | 160.7 |  | 159.3 |  |
| 3 | 6’ | 109.4 | 6.39 | 110.3 | 6.08 | 110.0 | 6.12 |
| 4 | 5’ |  |  | 185.3 |  |  |  |
| 4a | 4’a | 106.4 |  | 106.6 |  | 111.2 |  |
| 5 | 4’ | 156.3 |  | 157.3 |  | 157.2 |  |
| 5a | 3’a |  |  | 122.9 |  |  |  |
| 6 | 3 | 38.6 | 3.51 | 38.9 | 3.48 | 102.8 | 7.04 |
| 6 | 3 | 38.6 | 3.63 | 38.9 | 3.60 |  |  |
| 6a | 2’ | 113.0 |  | 112.6 |  |  |  |
| 7 | 3 | 28.2 | 2.40 | 28.4 | 2.35 | 38.1 | 1.73 |
| 7 | 3 | 28.2 | 2.58 | 28.4 | 2.55 |  |  |
| 8 | 4 | 21.6 | 3.10 | 21.8 | 3.08 | 23.9 | 2.88 |
| 8 | 4 | 21.6 | 3.17 | 21.8 | 3.14 |  |  |
| 8a | 4a | 131.9 |  |  |  |  |  |
| 9 | 5 | 123.9 | 7.54 | 124.1 | 7.46 | 124.2 | 7.45 |
| 9a | 5a | 129.7 |  |  |  |  |  |
| 10 | 6 | 109.1 | 7.52 | 109.5 | 7.29 | 109.4 | 7.26 |
| 11 | 7 | 144.1 |  | 146.1 |  | 146.5 |  |
| 13a | 9a | 114.2 |  | 113.8 |  | 113.5 |  |
| 14 | 10 |  |  |  |  |  |  |
| 14a | 10a | 146.5 |  | 146.6 |  | 152.6 |  |
| 16a | 9’a |  |  | 153.1 |  |  |  |
| 17 | 9’ | 149.4 |  |  |  | 159.0 |  |
| 5-OH | 4’-OH |  | 11.94 |  | 11.85 |  |  |
| 17-OH | 9’-OH |  |  |  | 13.12 |  |  |
| 1’ | 11 | 159.8 |  | 162.4 |  | 162.2 |  |
| 2’ | - |  | 9.99 |  |  |  |  |
| 3’ | - | 104.3 |  | 113.2 |  | 112.7 |  |
| 5a’ | - | 150.8 |  | 151.8 |  | 151.1 |  |
| 6’ | - | 116.3 | 7.46 | 116.8 | 7.42 | 116.4 | 7.43 |
| 7’ | - | 132.8 | 7.70 | 133.3 | 7.66 | 132.9 | 7.67 |
| 8’ | - | 124.7 | 7.42 | 125.1 | 7.39 | 124.7 | 7.39 |
| 9’ | - | 123.8 | 7.86 | 124.5 | 7.86 | 124.2 | 7.87 |
| 9a’ | - | 116.5 |  |  |  |  |  |
| 10’ | - | 150.8 |  | 159.5 |  | 153.8 |  |
| 1’’ | 10’ | 56.9 | 3.88 | 57.5 | 3.88 | 56.4 | 3.79 |
| 2’’ | - |  |  |  |  | 56.5 | 3.98 |
| 3’’ | - |  |  |  |  | 56.6 | 3.90 |
| 4’’ | - |  |  |  |  | 60.8 | 3.74 |
| 5’’ | - |  |  |  |  | 61.0 | 3.81 |
| 6’’ | - | 61.1 | 3.72 | 61.4 | 3.53 | 61.2 | 3.67 |
| 7’’ | - | 60.3 | 4.19 | 60.5 | 4.23 | 60.1 | 4.24 |
| 8’’ | - |  |  | 37.8 | 3.21 | 37.4 | 3.21 |

(a) Recorded at 125 MHz, (b) Recorded at 500 MHz, HMBC correlations are presented from proton(s) to the indicated carbon, The respective signal of DMSO-d_6_ was used to reference the NMR spectra.


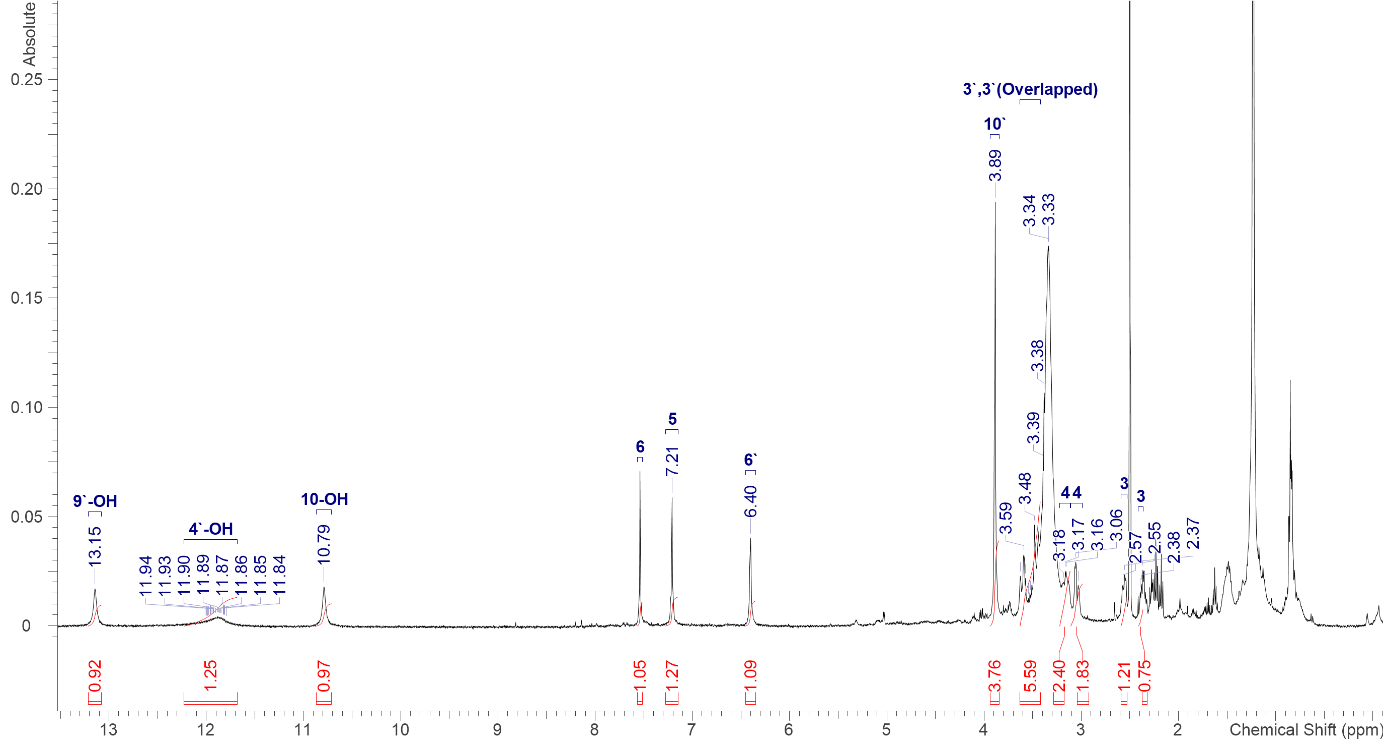


**Figure S16**: ^1^H NMR spectrum of rubromycin CA1 (**5**) (DMSO-d_6_, 500 MHz).


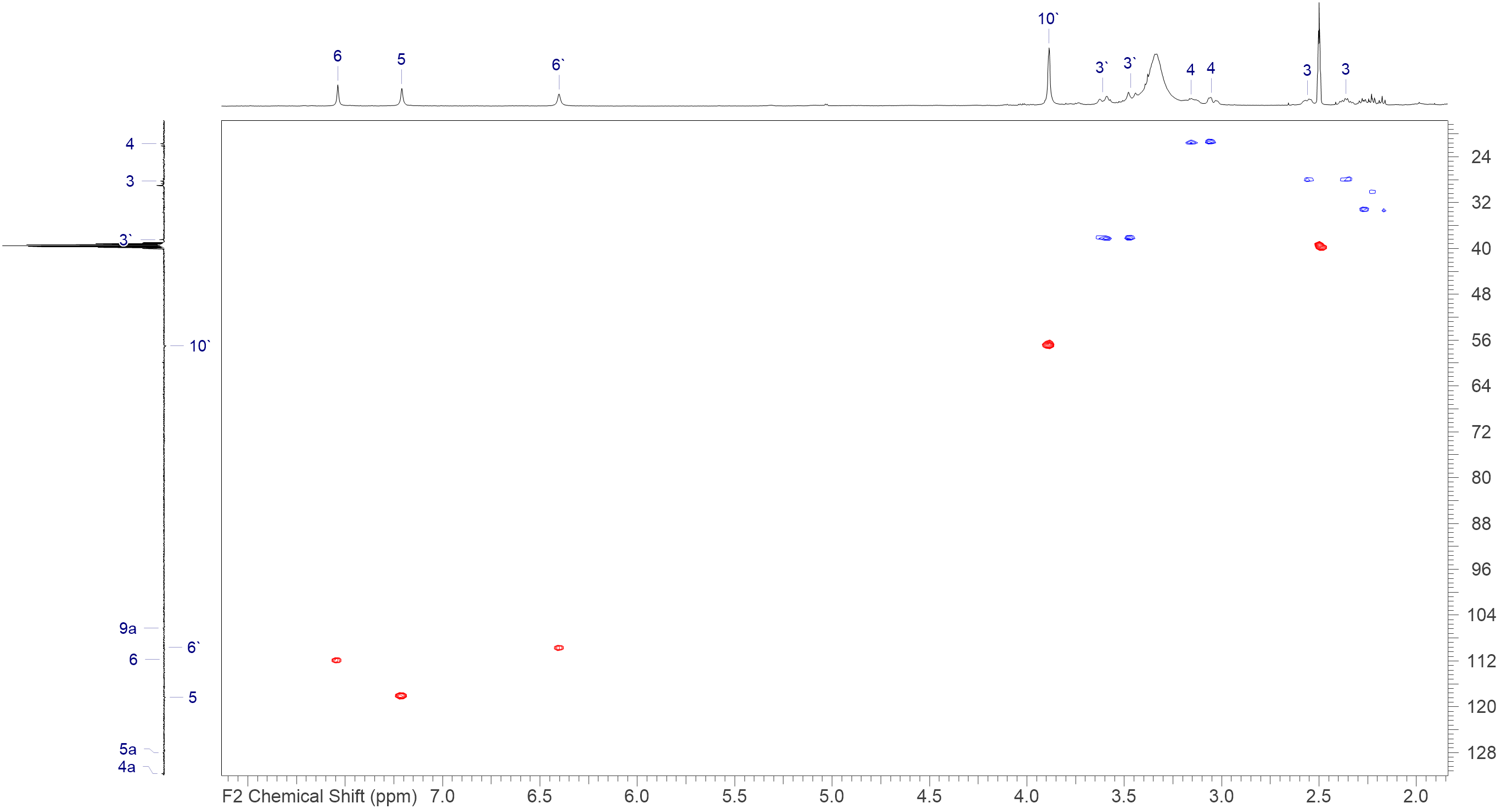


**Figure S17**: HSQC spectrum of rubromycin CA1 (**5**) (DMSO-d_6_, 500 MHz).


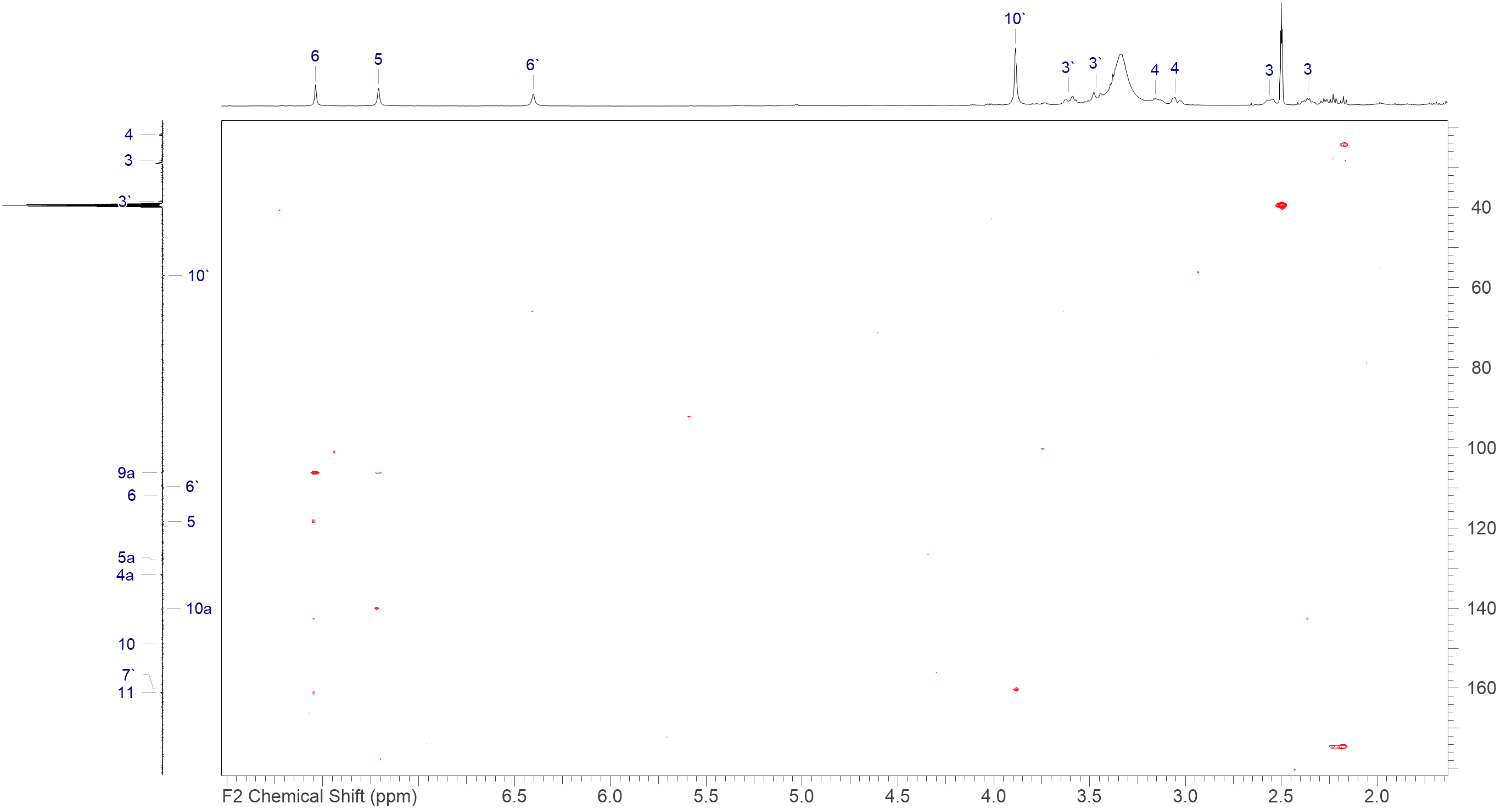


**Figure S18**: HMBC spectrum of rubromycin CA1 (**5**) (DMSO-d_6_, 500 MHz).


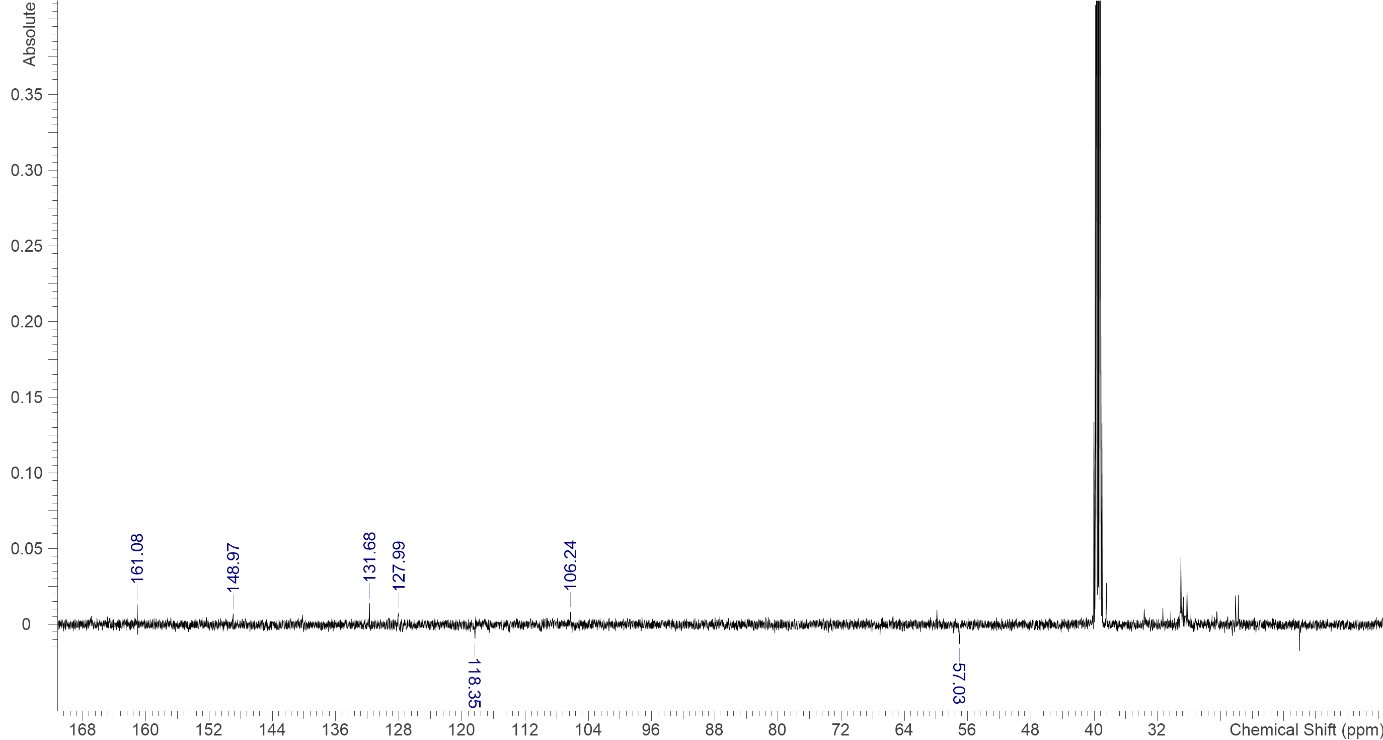


**Figure S19**: ^13^C-DEPTq NMR spectrum of rubromycin CA1 (**5**) (DMSO-d_6_, 125 MHz).


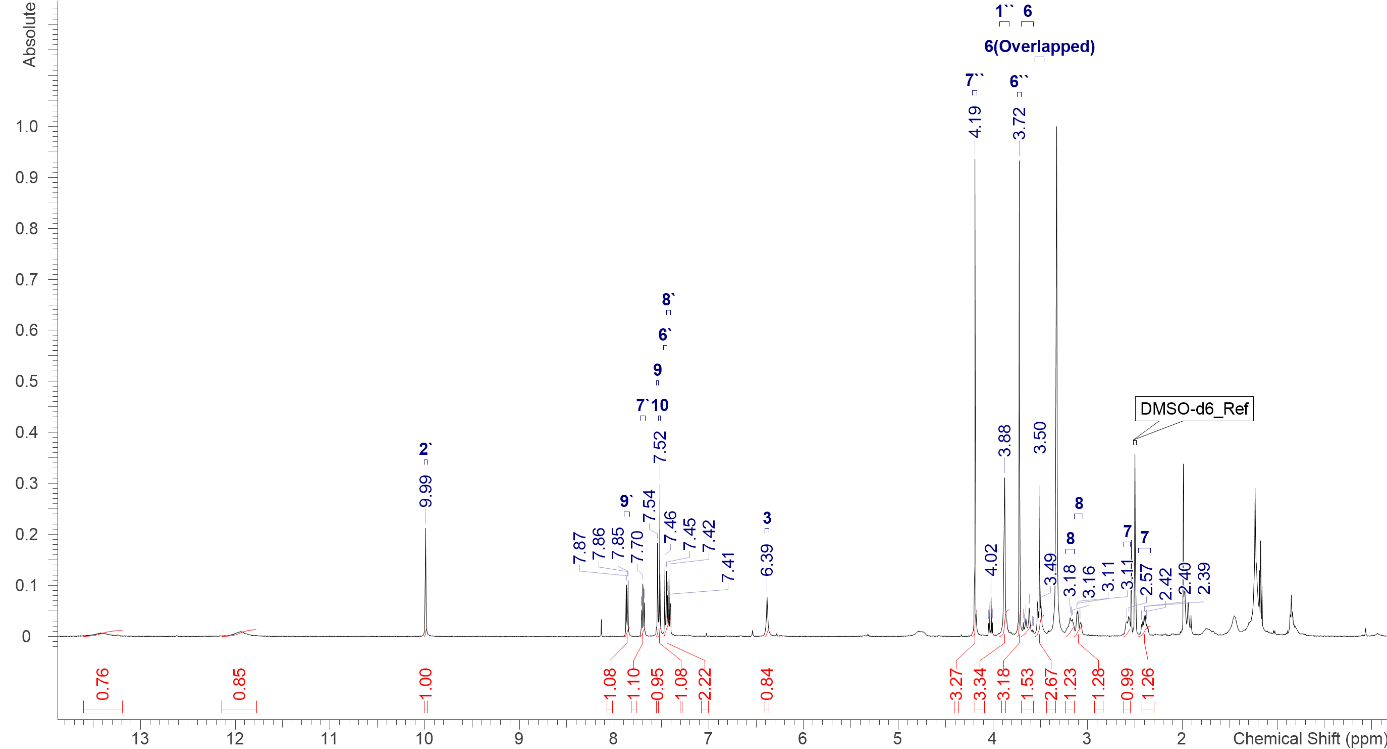


**Figure S20**: ^1^H NMR spectrum of 2xMet-coumarubrin (**8**) (DMSO-d_6_, 500 MHz).


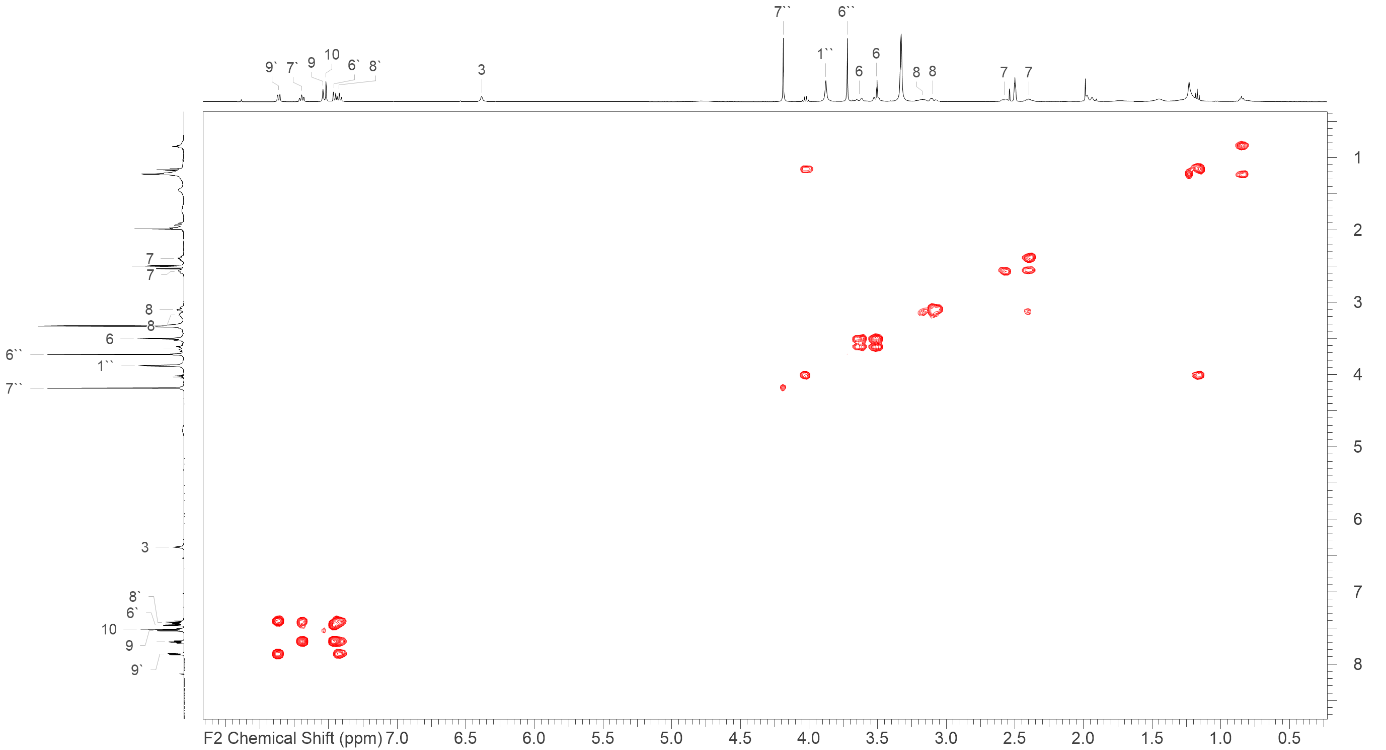


**Figure S21**: COSY spectrum of 2xMet-coumarubrin (**8**) (DMSO-d_6_, 500 MHz).


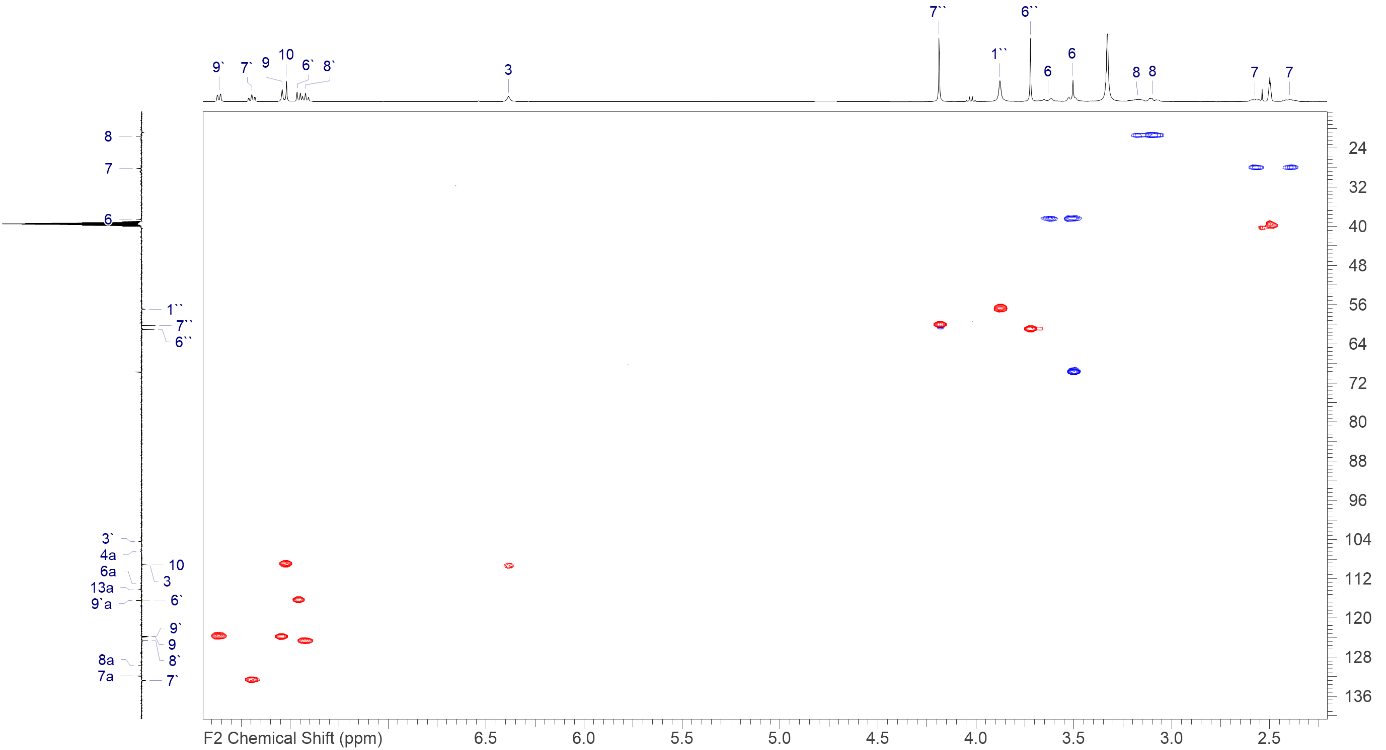


**Figure S22**: HSQC spectrum of 2xMet-coumarubrin (**8**) (DMSO-d_6_, 500 MHz).


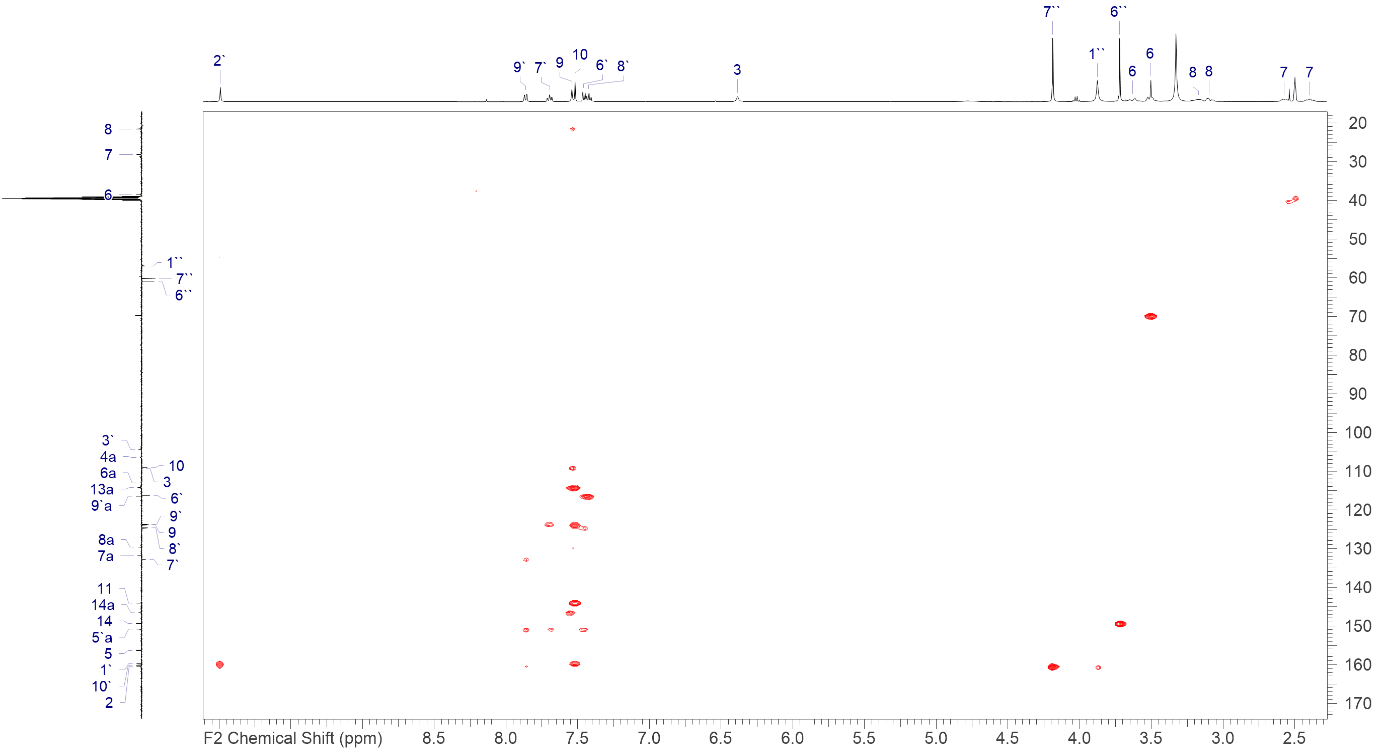


**Figure S23**: HMBC spectrum of 2xMet-coumarubrin (**8**) (DMSO-d_6_, 500 MHz).


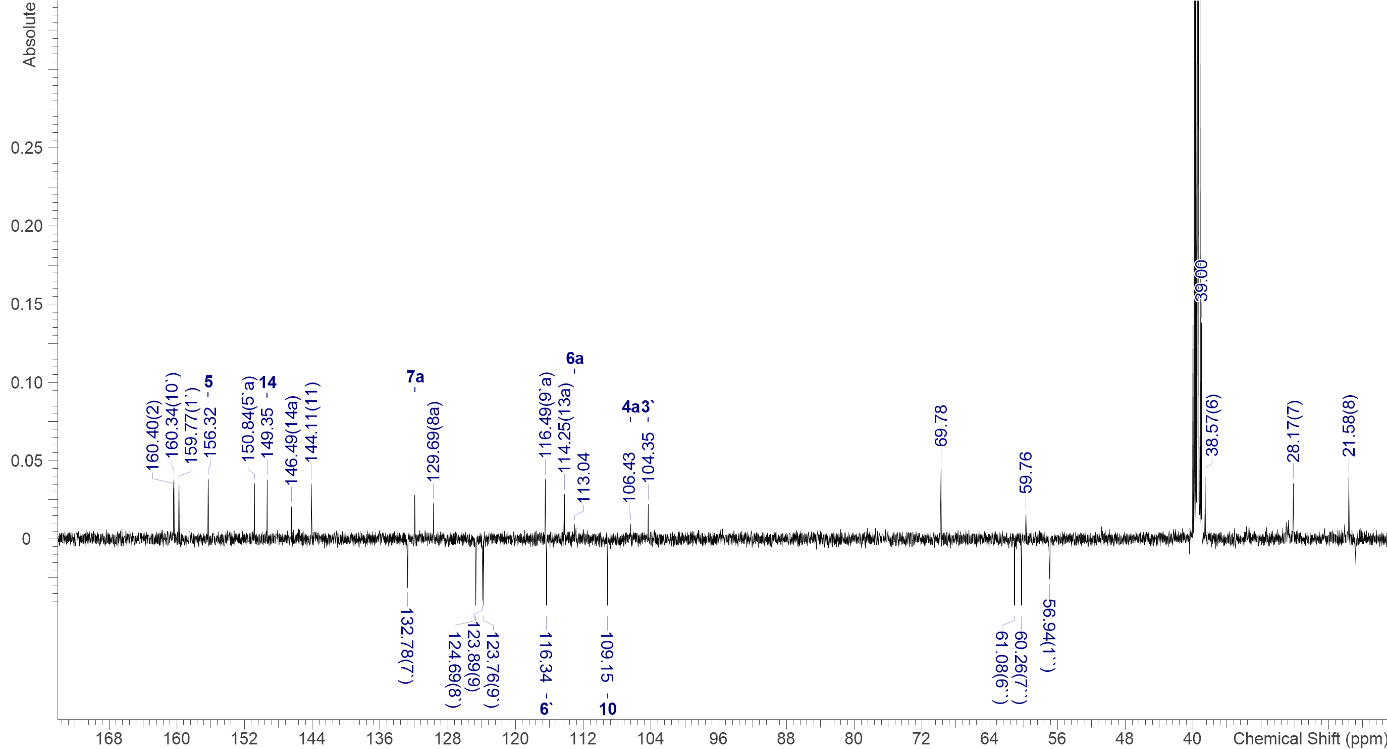


**Figure S24**: ^13^C-DEPTq NMR spectrum of 2xMet-coumarubrin (**8**) (DMSO-d_6_, 125 MHz).


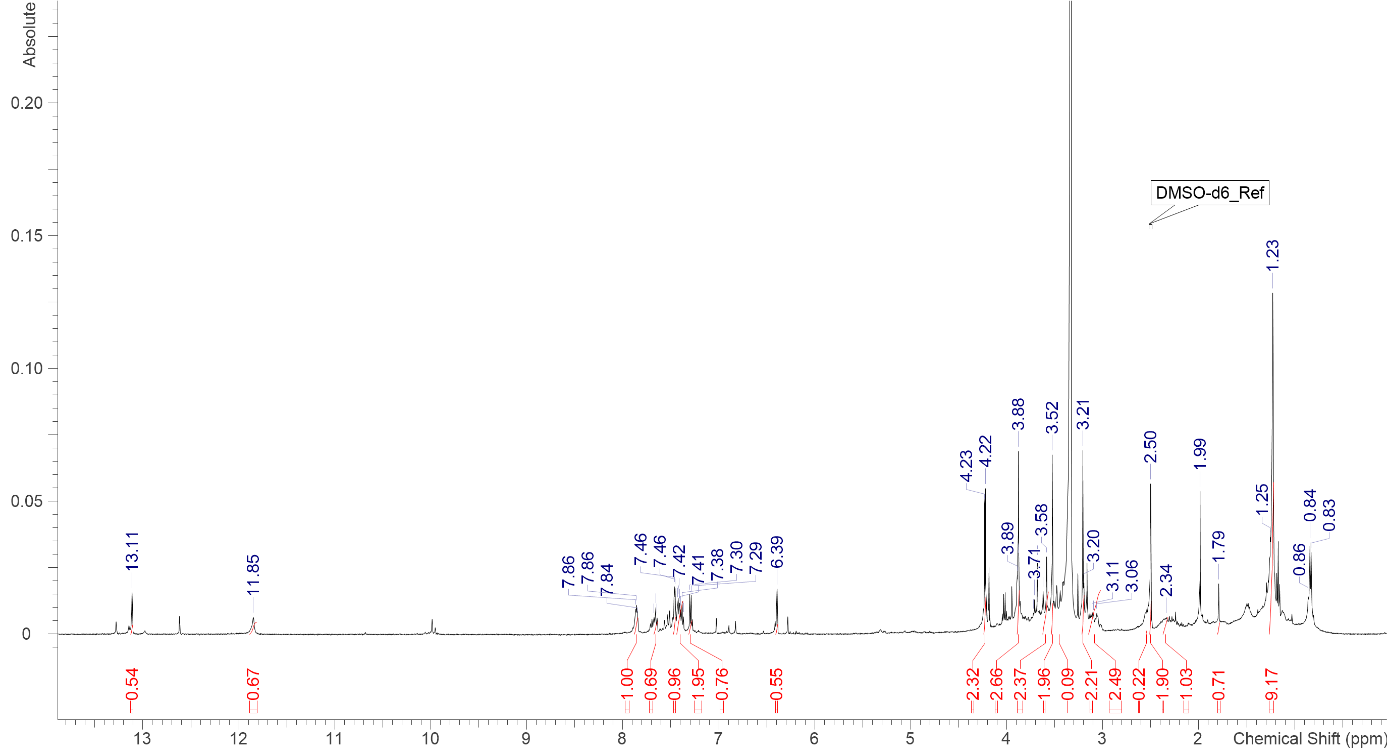


**Figure S25**: ^1^H NMR spectrum of 3xMet-coumarubrin (**9**) (DMSO-d_6_, 500 MHz).


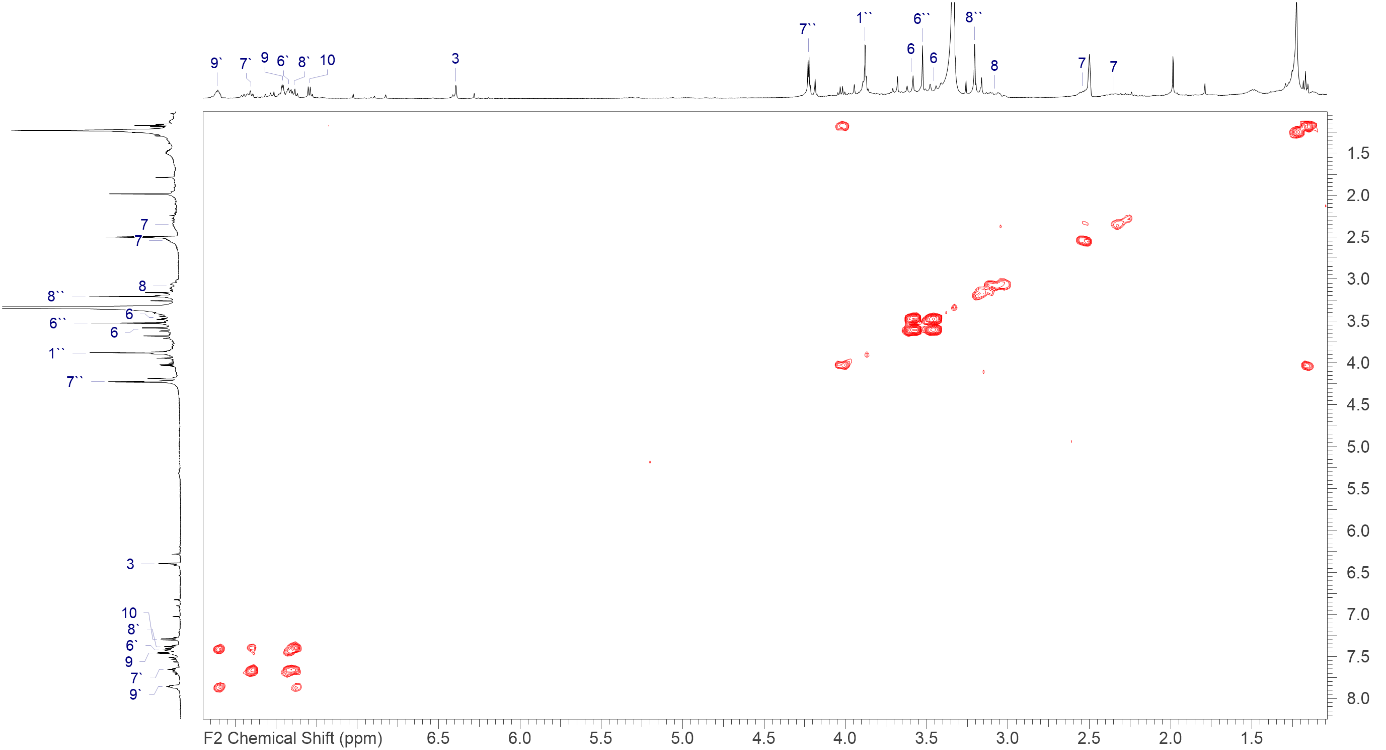


**Figure S26**: COSY spectrum of 3xMet-coumarubrin (**9**) (DMSO-d_6_, 500 MHz).


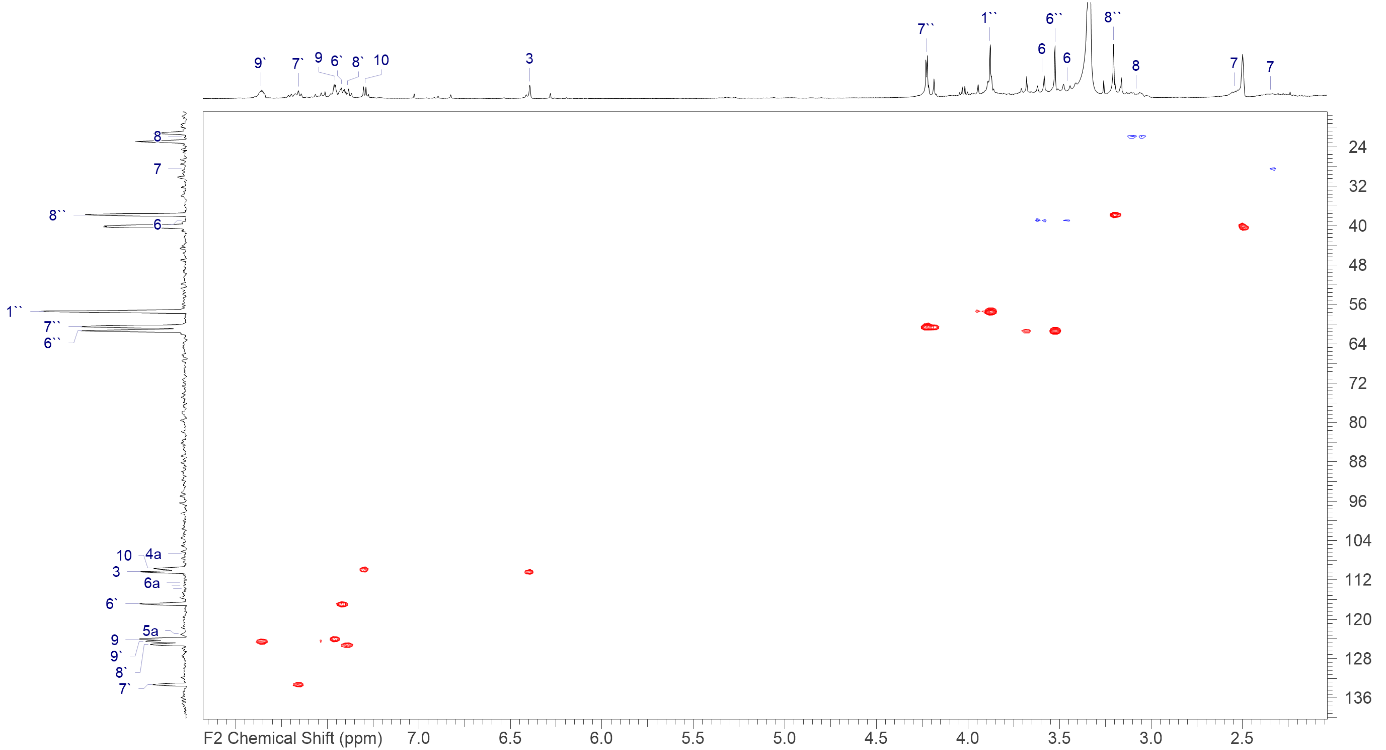


**Figure S27**: HSQC spectrum of 3xMet-coumarubrin (**9**) (DMSO-d_6_, 500 MHz).


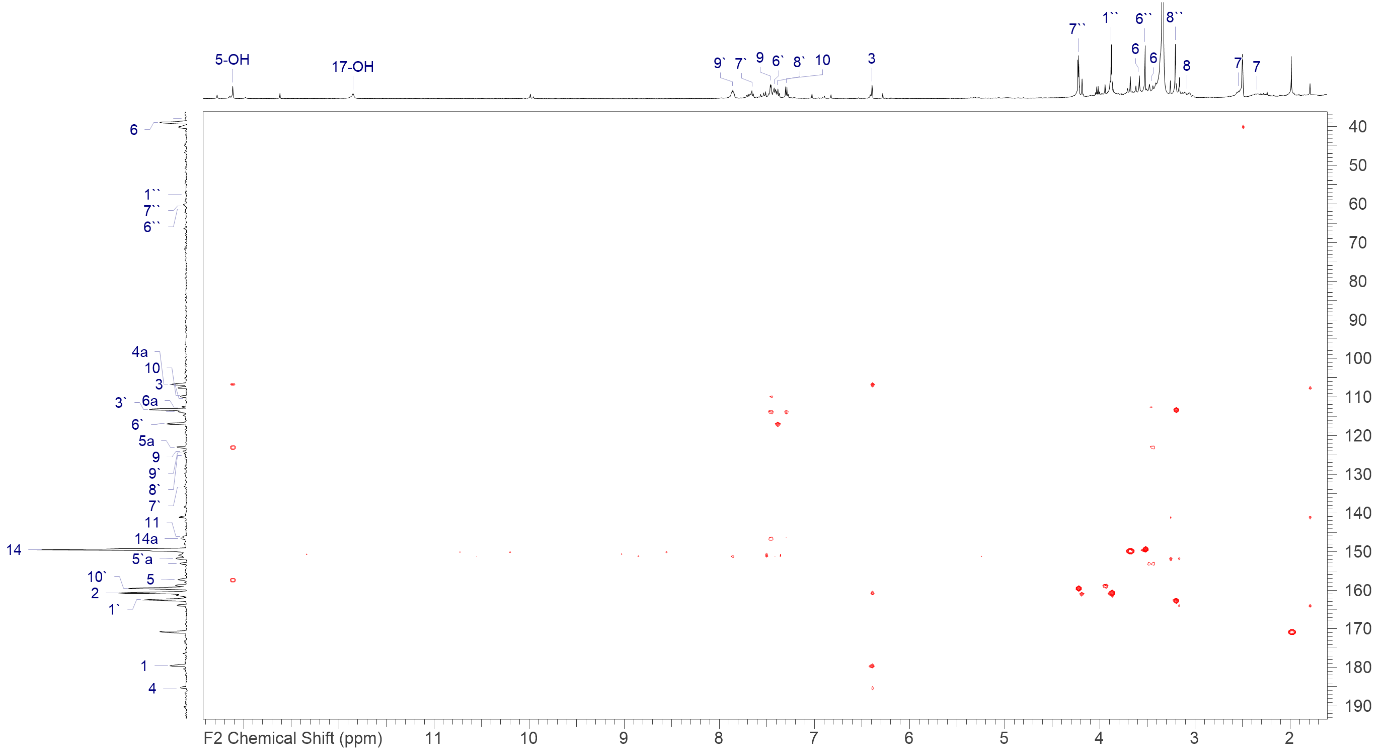


**Figure S28**: HMBC spectrum of 3xMet-coumarubrin (**9**) (DMSO-d_6_, 500 MHz).


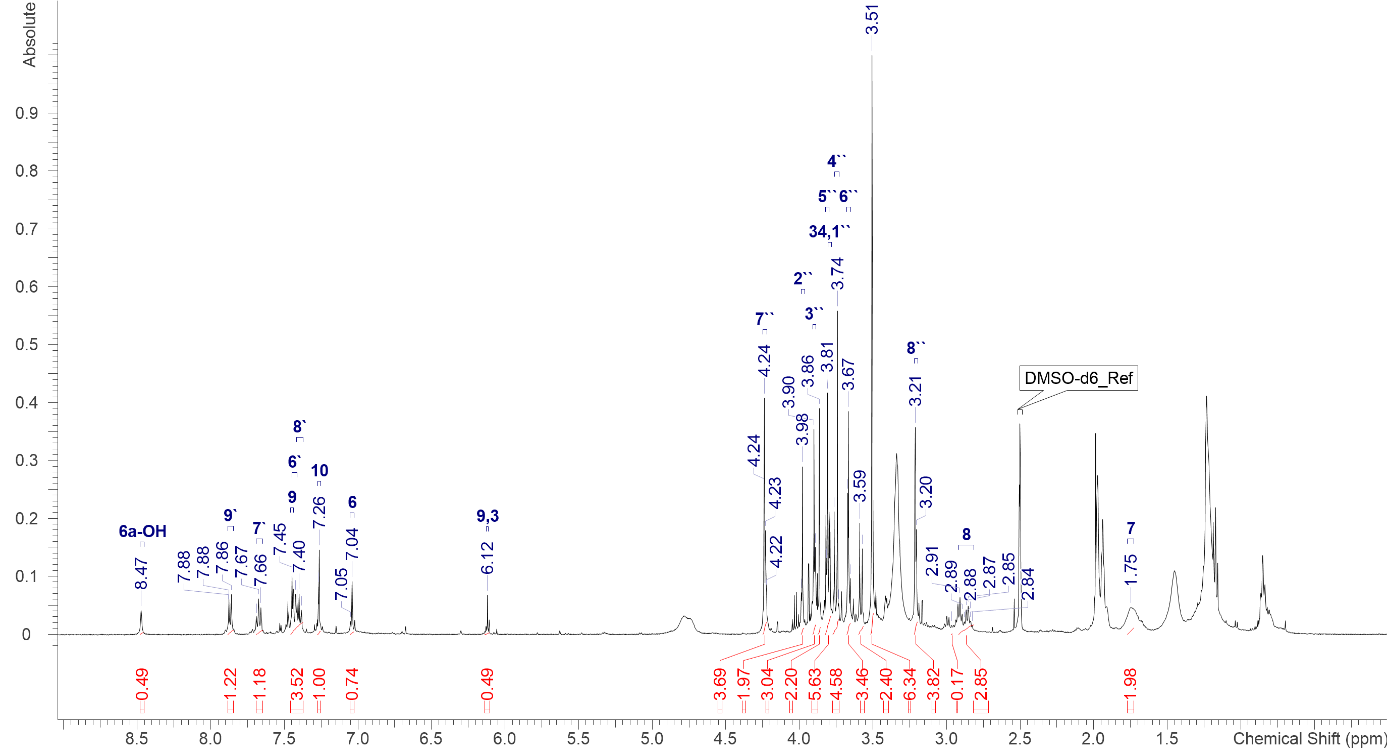


**Figure S29**: ^1^H NMR spectrum of 7xMet-coumarubrin (**10**) (DMSO-d_6_, 500 MHz).


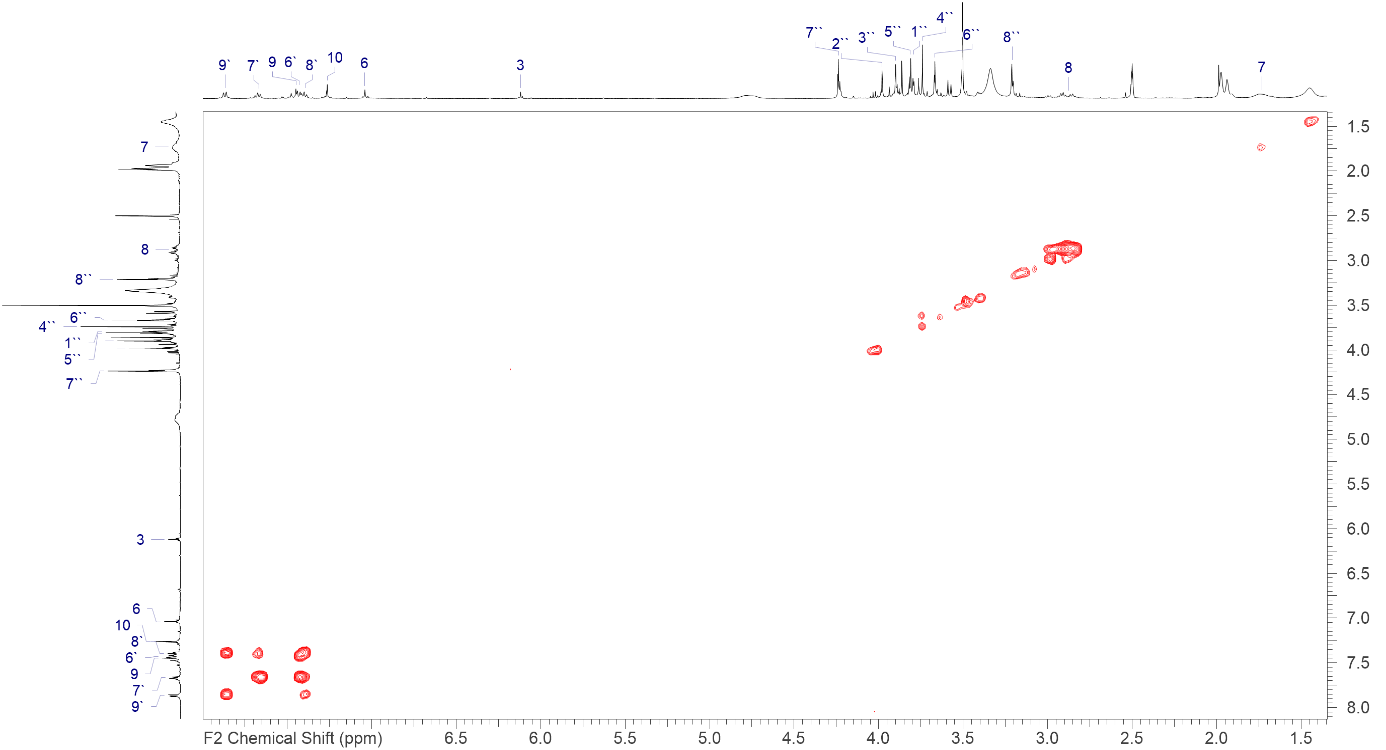


**Figure S30**: COSY spectrum of 7xMet-coumarubrin (**10**) (DMSO-d_6_, 500 MHz).


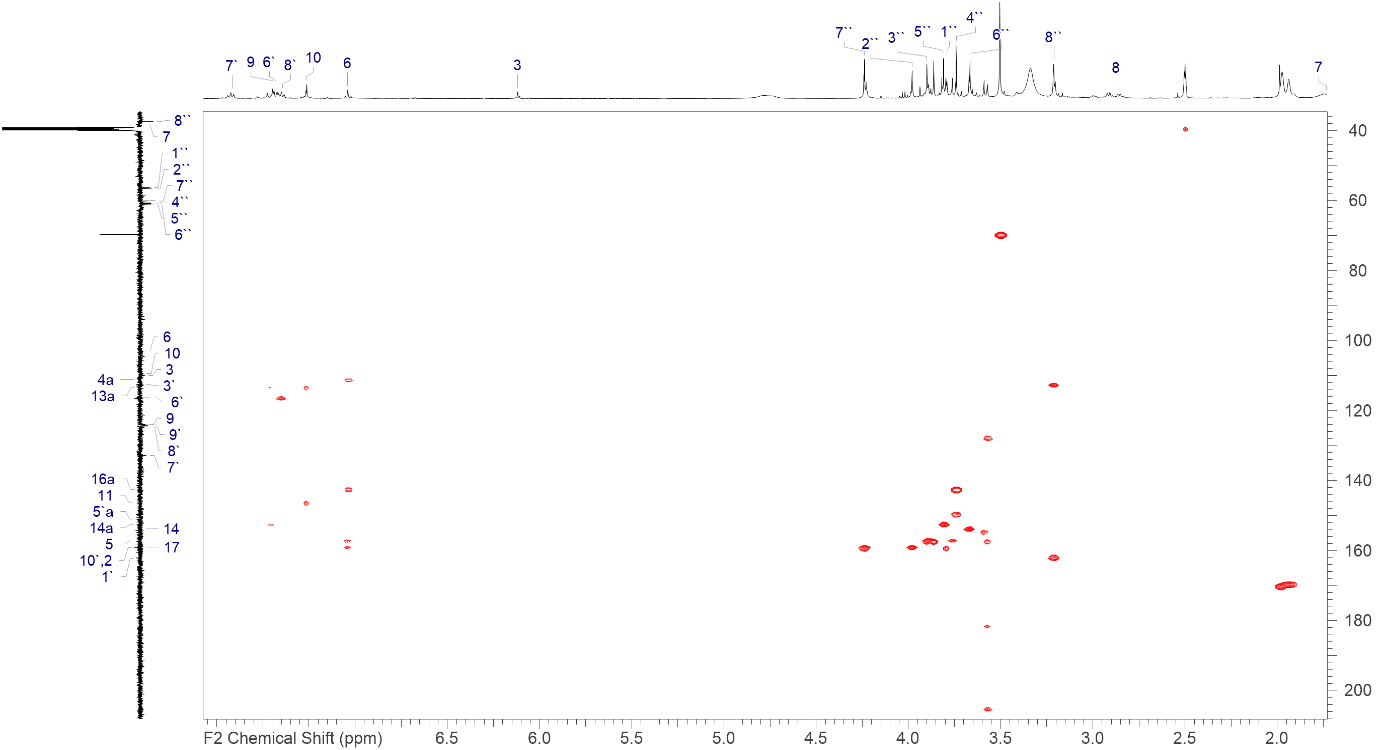


**Figure S31**: HSQC spectrum of 7xMet-coumarubrin (**10**) (DMSO-d_6_, 500 MHz).


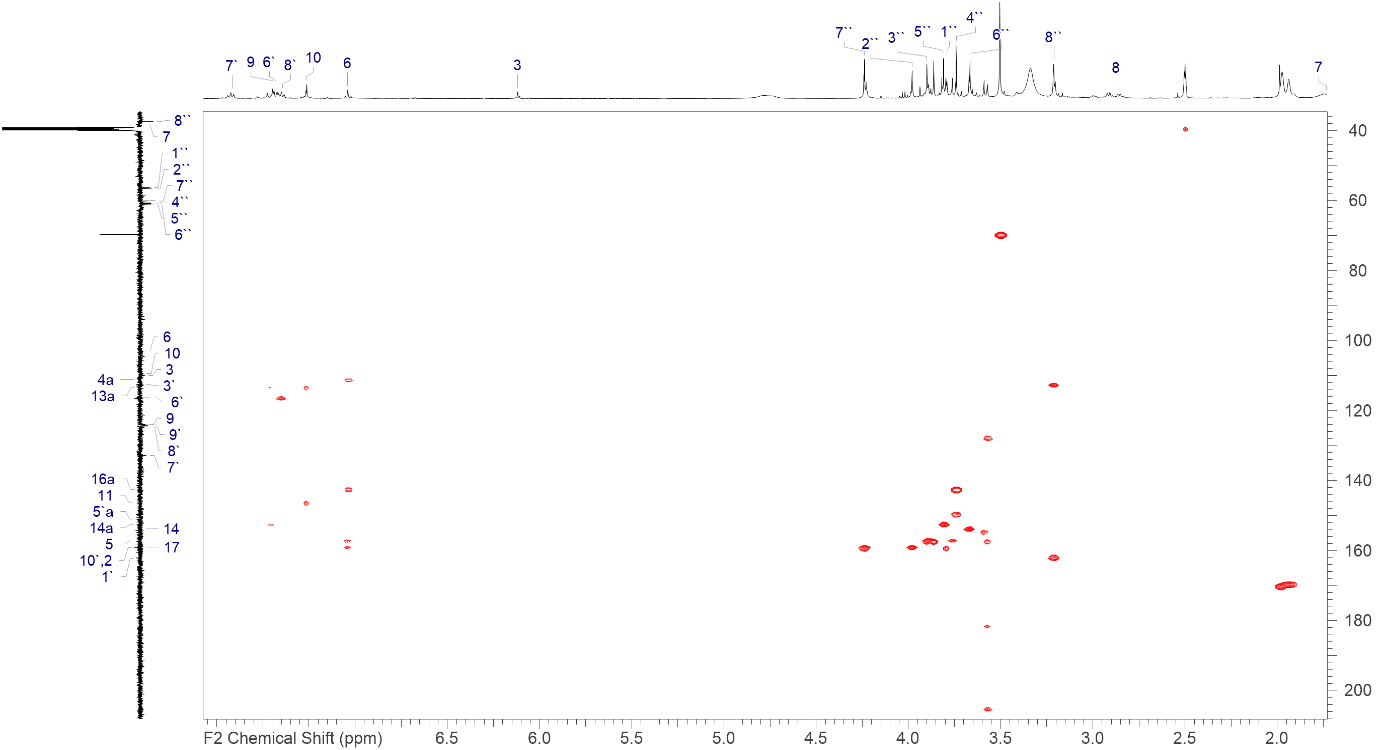


**Figure S32**: HMBC spectrum of 7xMet-coumarubrin (**10**) (DMSO-d_6_, 500 MHz).


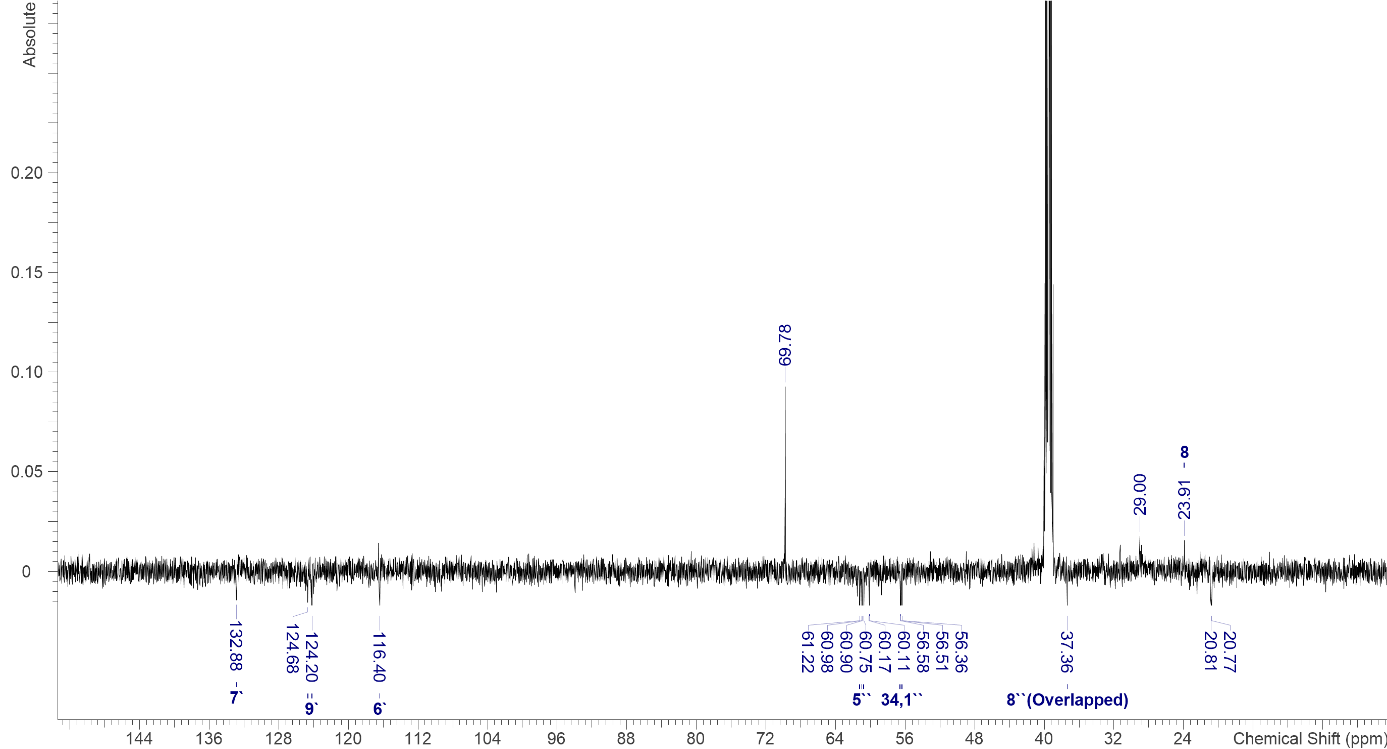


**Figure S33**: ^13^C-DEPTq NMR spectrum of 7xMet-coumarubrin (**10**) (DMSO-d_6_, 125 MHz).


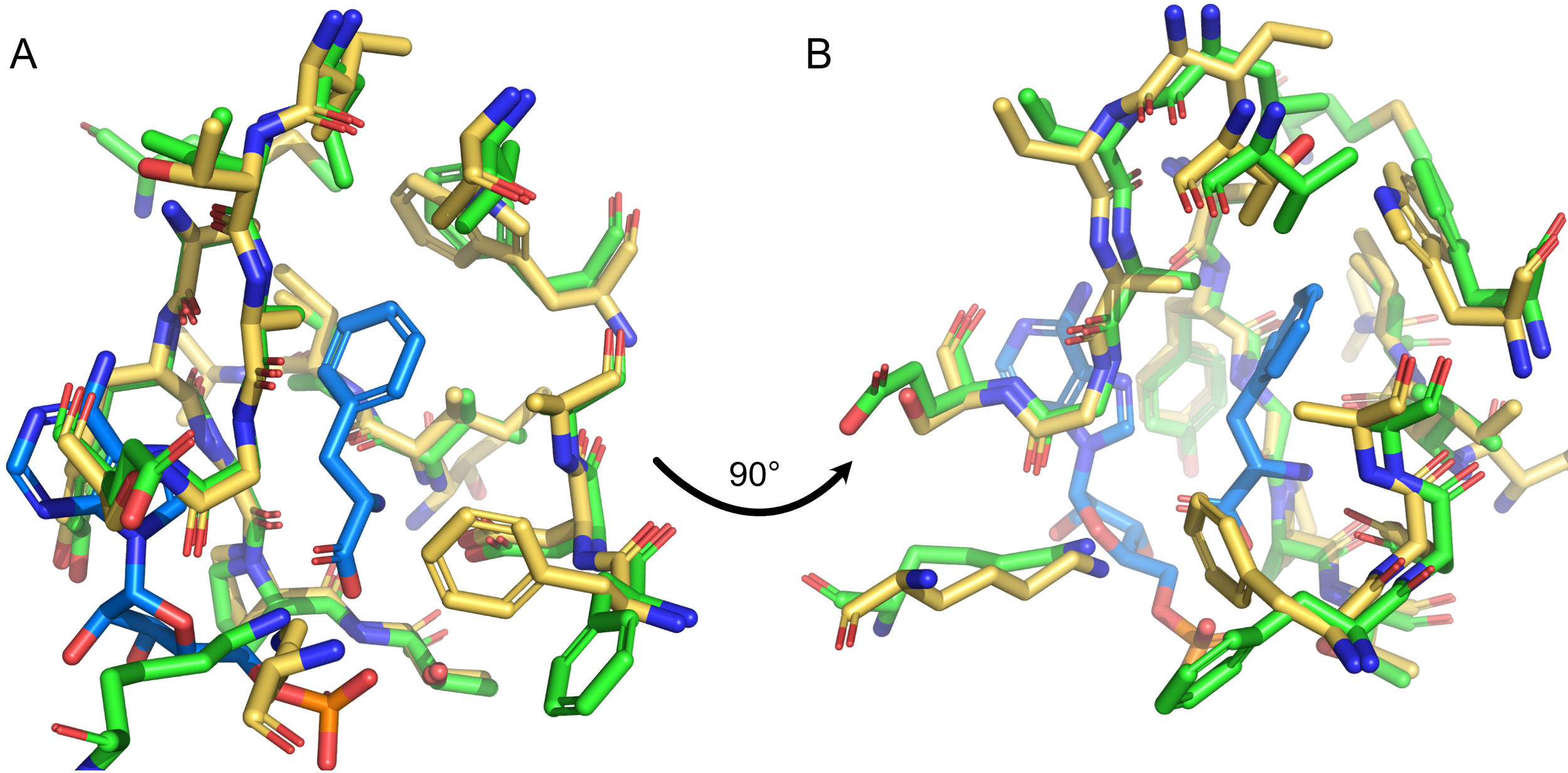


**Figure S34**: Representation of the structural alignment of the phenylalanine activating domain of the gramicidin synthetase 1 (pdb ID 1AMU, yellow-orange sticks) in complex with AMP and phenylalanine (marine) overlaid with the AlphaFold3 model of the NRPS-like NovH homolog (WP_222598543.1) from the *L. tibetensis* rubromycin BGC. The structural alignment is represented from two orientations highlighting the virtually identical phenylalanine binding site.

**Supplemental References**

Frensch, B., Lechtenberg, T., Kather, M., Yunt, Z., Betschart, M., Kammerer, B., Ludeke, S., Muller, M., Piel, J., & Teufel, R. (2021). Enzymatic spiroketal formation via oxidative rearrangement of pentangular polyketides. *Nat Commun*, *12*(1), 1431. <https://doi.org/10.1038/s41467-021-21432-9>

Harunari, E., Imada, C., & Igarashi, Y. (2019). Konamycins A and B and Rubromycins CA1 and CA2, Aromatic Polyketides from the Tunicate-Derived Streptomyces hyaluromycini MB-PO13(T). *J Nat Prod*, *82*(6), 1609-1615. <https://doi.org/10.1021/acs.jnatprod.9b00107>

Toplak, M., Nagel, A., Frensch, B., Lechtenberg, T., & Teufel, R. (2022). An acetyltransferase controls the metabolic flux in rubromycin polyketide biosynthesis by direct modulation of redox tailoring enzymes. *Chem Sci*, *13*(24), 7157-7164. <https://doi.org/10.1039/d2sc01952c>

Yi, L. W., Yi, S. R., Wang, Y. J., Kong, J. Q., Xiong, Y., Zhou, Y. S., Duan, Y. W., & Zhu, X. C. (2024). Late-Stage Tailoring Steps in the Biosynthesis of β-Rubromycin Involve Successive Terminal Oxidations, a Selective Hydroxyl Reduction, and Distinctive O-Methylations. *Org Lett*, *27*(1), 103-108. <https://doi.org/10.1021/acs.orglett.4c03965>
